# Soft Artificial Ciliary Brush with Integrated Haptic Feedback for Efficient Airway Mucus Cleaning

**DOI:** 10.1002/aisy.202501236

**Published:** 2026-01-06

**Authors:** Zhongming Lyu, Yusheng Wang, Ruijian Ge, Darren Wang, Matthew Bacchetta, Caitlin T. Demarest, Fabien Maldonado, Xiaoguang Dong

**Affiliations:** Department of Mechanical Engineering, Vanderbilt University, Nashville 37212, TN, US; Department of Mechanical Engineering, Vanderbilt University, Nashville 37212, TN, US; Department of Mechanical Engineering, Vanderbilt University, Nashville 37212, TN, US; Department of Mechanical Engineering, Vanderbilt University, Nashville 37212, TN, US; Division of Allergy, Pulmonary and Critical Care Medicine, School of Medicine, Vanderbilt University, Nashville, TN 37232, US; Department of Thoracic Surgery, Vanderbilt University Medical Center, Nashville, TN 37232, US; Division of Allergy, Pulmonary and Critical Care Medicine, School of Medicine, Vanderbilt University, Nashville, TN 37232, US; Department of Mechanical Engineering, Vanderbilt University, Nashville 37212, TN, US; Department of Mechanical Engineering, Vanderbilt University, Nashville 37212, TN, US; Department of Biomedical Engineering, Vanderbilt University, Nashville 37212, TN, US; Vanderbilt Institute for Surgery and Engineering, Vanderbilt University, Nashville 37212, TN, US

**Keywords:** airway plugging, artificial cilia, haptic sensing, mucus cleaning, soft robot

## Abstract

Mucus accumulation is a major complication in cystic fibrosis (CF) and chronic obstructive pulmonary disease (COPD), increasing risks of airway obstruction and impairing drug delivery. Maintaining the patency of airway prosthetic devices such as stents and endotracheal tubes also requires effective clearance. Current approaches, primarily blind suction, are ineffective against viscous mucus and risk tissue trauma. Here, it proposes a mucus-cleaning catheter that integrates a rotational soft ciliary brush with suction for efficient and safe clearance on tracheobronchial tissues and within airway prosthetic devices. The soft artificial cilia efficiently wrap up mucus around the suction inlets for removing viscous mucus while a haptic sensing unit provides real-time feedback for operational safety. The device can remove mucus in thin layers without damaging the tissue, typically difficult for pure suction by a therapeutic bronchoscope. This study demonstrates that the device can access airway phantom models, ex vivo ovine lungs, airway stents, and endotracheal tubes, performing on-demand mucus clearance under real-time visualization via an onboard camera. Compared with therapeutic bronchoscopes with pure suction, the catheter provides faster clearance and minimal damage to tissues, offering a promising strategy for safe, efficient, and timely mucus cleaning for patients with excessive mucus accumulation in the airway.

## 1. Introduction

Mucus plugging in the trachea or within airway stents increases the risk of inflammation, as the thickened mucus traps bacteria and other pathogens, promoting recurrent infections and chronic inflammation that damages lung tissue and exacerbates mucus abnormalities.^[1]^ First, excessive mucus accumulation in the airway is common in several lung diseases including chronic obstructive pulmonary diseases (COPD),^[2]^ and cystic fibrosis (CF).^[3]^ Particularly, in CF, airway mucus is abnormally thick, sticky, and dehydrated due to a genetic mutation in the cystic fibrosis transmembrane conductance regulator (CFTR) gene,^[4,5]^ which disrupts the balance of salt and water in airway secretions. This leads to impaired mucociliary clearance, where the cilia cannot effectively mobilize mucus out of the lungs,^[6]^ resulting in its accumulation. These changes cause significant airway obstruction, making it difficult to breathe and interfere with gas exchange, and promoting infections. Therefore, the combination of airway obstruction, infection, and inflammation leads to progressive lung damage, including bronchiectasis and respiratory failure.^[7]^ The thick, sticky nature of mucus also poses a major barrier to drug delivery by restricting diffusion and trapping therapeutic agents at its surface. Clearing this mucus barrier remains a critical challenge for effective therapeutic delivery. In addition, excessive mucus accumulation also occurs in airway prosthetic devices such as endotracheal tubes^[8]^ and airway stents,^[9,10]^ which are hollow structures designed to support the airway during stricture and assist patients in breathing. For instance, airway stents are commonly made of silicone or mesh-covered metal to prevent tissue ingrowth,^[11]^ but these materials impede normal mucociliary clearance. Patients with implanted stents often experience mucus plugging within the device.

To reduce the mucus plugging and improve airway function, various airway clearance techniques (ACTs) have been developed. First, chest physiotherapy, positive expiratory pressure (PEP) devices, and high-frequency chest wall oscillation^[12,13]^ have been proposed aiming to physically loosen and mobilize mucus. Mucolytic medicines have been reported but are inefficient in thinning and mobilizing viscous mucus.^[14]^ These methods primarily reduce mucus layer thickness and alleviate obstruction without efficiently eliminating the mucus barrier itself. In more severe cases, endotracheal suction^[15]^ during intubation and mechanical ventilation are employed for clearing mucus. Currently, on-demand mucus removal remains limited to blind suction techniques using mucus extractors,^[16]^ specialized endotracheal tubes,^[17,18]^ or catheters with suction tubes designed for mucus clearance.^[19,20]^ However, these tools show limited cleaning performance due to the simple mucus cleaning mechanism relying on only suction or mechanical scratching, especially when the mucus gets viscous. There is also lack of sensory feedback to avoid tissue damage. Existing blind suctions with relatively rigid tips may cause significant suction trauma. So far, it remains challenging to remove viscous mucus in the airway or inside airway prosthetic devices.

On the other hand, steerable catheters have been developed to access the airway for applications such as bronchoscope–ultrasound imaging,^[21]^ optical coherence tomography,^[22]^ and targeted biopsy. Reported steering strategies include tendon-driven mechanisms,^[23]^ push–pull actuation based on notched concentric tubes,^[24]^ and alternative methods, such as pneumatic actuation^[22]^ or magnetic steering.^[25]^

However, mucus cleaning using a steerable catheter has not been demonstrated.

To tackle this challenge, we present a mucus cleaning mechanism by integrating both a rotational soft artificial ciliary brush and suction to efficiently and safely clean mucus on tracheobronchial tissues and airway medical devices. First, the soft artificial cilia efficiently wrap up mucus around the suction inlets for removing viscous mucus. We systematically optimize the design of the cleaning head in terms of geometry, size, and artificial cilia distribution. The device can remove mucus in thin layers without damaging the tissue, typically difficult for pure suction by a therapeutic bronchoscope. Second, a haptic sensing unit based on magnetic sensing is integrated which provides real-time feedback for operational safety. We systematically evaluated the contact force and evaluated the accuracy and safety. Finally, we demonstrate that a steerable catheter integrating mucus-cleaning and haptic-sensing units can navigate the trachea and bronchi in both phantom models and ex vivo ovine lungs. The device also operates effectively within airway stents and endotracheal tubes, enabling on-demand mucus removal under real-time visualization provided by a miniature onboard camera. Compared with therapeutic bronchoscopes with pure suction, the catheter provides faster clearance and minimal damage to tissues. The proposed device offers a promising strategy for safe, efficient, and timely mucus cleaning for patients with excessive mucus accumulation in the airway.

## 2. Results

### 2.1. Overall Concept and Design of the Mucus Cleaning Catheter with Haptic Feedback

We present a catheter system featuring a cleaning head that integrates a soft ciliary brush with suction to enhance airway mucus clearance. While bioinspired cilia^[26]^ have previously been reported to pump low-viscosity fluids in lab-on-a-chip devices,^[27-30]^ their application to mucus clearance remains highly challenging due to the fluid’s high viscosity. To address this, a soft ciliary brush is introduced, which can be rotated either directly by a motor or via a mechanical transmission, enabling sufficiently strong actuation for effective removal of viscous mucus.

**Figure 1.**
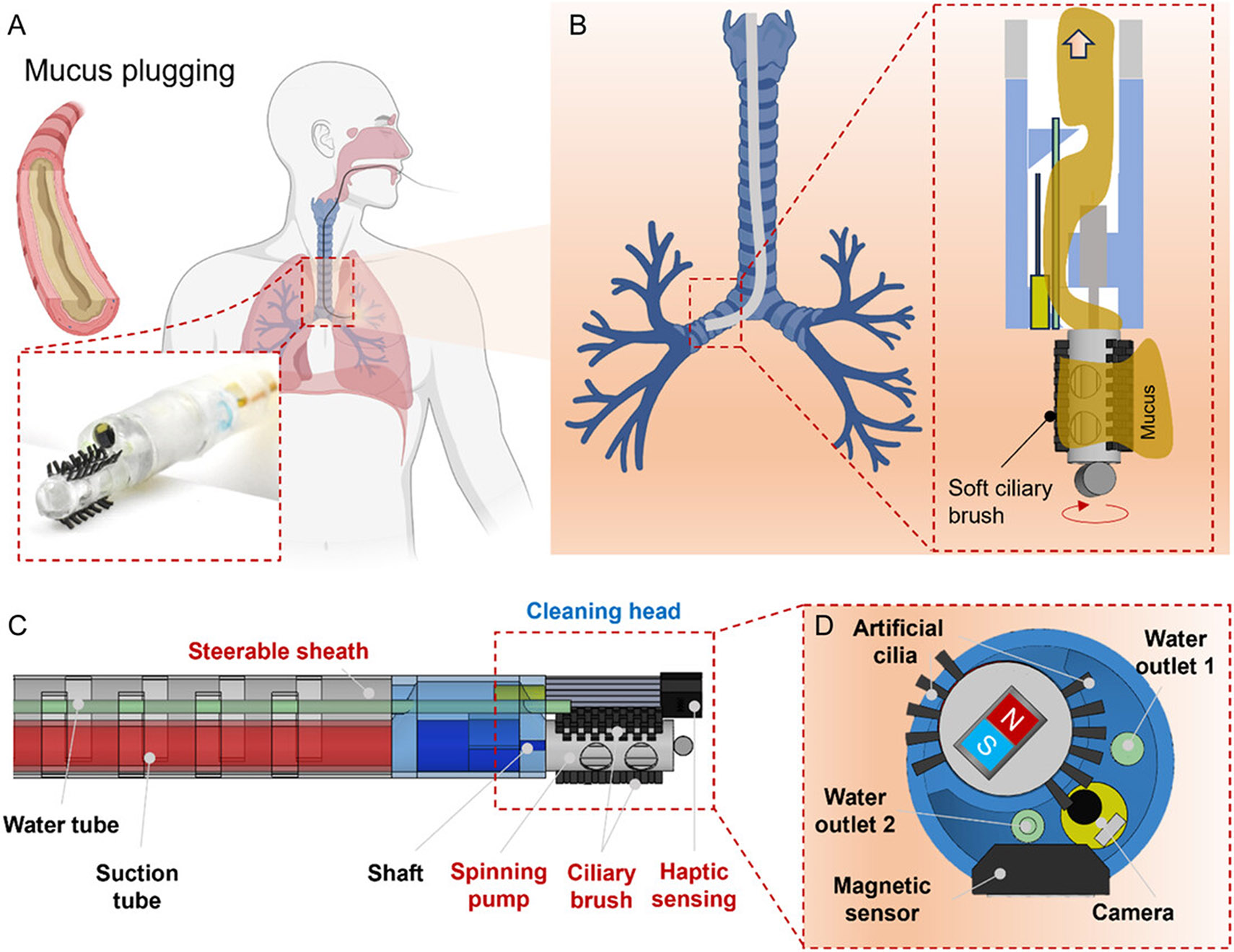
Concept and overall design of the mucus-cleaning catheter integrating soft artificial cilia and haptic feedback. A) Schematic illustration of the steerable catheter system designed for mucus removal in airway environments. Created by biorender.com. B) Illustration of the soft ciliary brush and spinning pump for cleaning mucus inside the airway. Created by biorender.com. C) 3D rendering of the fully assembled mucus-cleaning device integrating the proposed cleaning head. The outer diameter of the catheter is 8 mm as a proof-of-concept demonstration and can be scaled down to 6 mm. The catheter body with a length of 50 mm is made of thermoplastic polyurethane (TPU), while the sheath with notched features is fabricated from Elastic 50A (Formlabs Inc.). D) Side view of the cleaning head with the integrated artificial cilia, the magnetic marker and sensor for sensing contact for providing haptic feedback.

As shown in Figure 1A, the catheter has a diameter of 8 mm as a proof-of-concept and can navigate the trachea and bronchi areas under visual guidance provided by an integrated miniature camera with a diameter of 2 mm. The core innovation lies in the cleaning head, which incorporates a soft ciliary brush made of soft beam structure, termed artificial cilia, designed to mechanically mobilize and collect mucus. The soft ciliary brush is bonded to a spinning pump which has an outer diameter of 4 mm and an inner diameter of 3.5 mm with hollow internal structures and holes on the surface for suction (Figure S1, Supporting Information). The soft ciliary brush features five artificial cilia arrays on each side, inlet holes of 2.5 mm in diameter, and a funnel structure with an outer diameter (OD) of 8 mm and inner diameter (ID) of 7 mm. Each artificial cilium is ≈1 mm long, with seven or eight artificial cilia per row (Figure S2, Supporting Information). Made from soft elastomer, the artificial cilia minimize potential damage to surrounding tissues. Moreover, four suction holes with 2.5 mm in diameter are distributed along the cylinder surface, connected via a funnel at the motor site to a suction tube that generates negative pressure for mucus removal. Unlike conventional suction-based methods, which only remove mucus in direct contact with the inlet, the artificial cilia actively sweep nearby mucus toward the cleaning head (Figure S3, Supporting Information), where it is subsequently evacuated by suction through the inlet holes. This dual-mode mechanism with the mechanical sweeping combined with suction, enables more thorough and efficient airway cleaning (Figure 1B) compared with using suction or mechanical sweeping alone.

Figures 1C illustrates the system components of the catheter implemented with the cleaning mechanism. The catheter has a steerable sheath for steering and the cleaning head for mucus cleaning. The steering is achieved by incorporating arrays of notches along the catheter body that facilitate tendon-driven actuation of both the catheter and the cleaning head. The cleaning head is equipped with a miniature camera, water tubing outlets, a spinning pump with a ciliary brush, and a magnetic sensor. The spinning pump is a cylindrical structure (OD: 4 mm) with an internal hollow channel (ID, 2.0 mm; OD, 3.5 mm). It is mounted on a miniature DC motor (OD: 4 mm) via a shaft fixed to a central hollow core (ID: 1 mm inner). To remove highly viscous mucus, such as in severe cystic fibrosis, a water channel can deliver saline or water to dilute secretions, thereby improving pump and suction efficiency (Figure S4, Supporting Information).

Another key feature is the haptic feedback system (Figure 1D), designed to prevent tissue damage and motor stalls. A cylindrical magnet (OD, 1.5 mm; length, 2.5 mm) is bonded to the spinning pump, while a magnetic sensor mounted on the side of the cleaning head detects periodic changes in the magnetic field to measure the spinning speed. As the cleaning head approaches tissue, friction slows its rotation, providing real-time feedback for controlled operation at safe distances. To further enhance visualization, a miniature camera (OD: 2 mm) is integrated at the tip of the spinning pump, for monitoring the surrounding tissue environment. In addition, a water outlet (OD, 1 mm; ID, 0.5 mm) is incorporated for ejecting water to clean the camera lens on demand.

### 2.2. Design and Optimization of the Cleaning Head for Effective Mucus Cleaning

**Figure 2.**
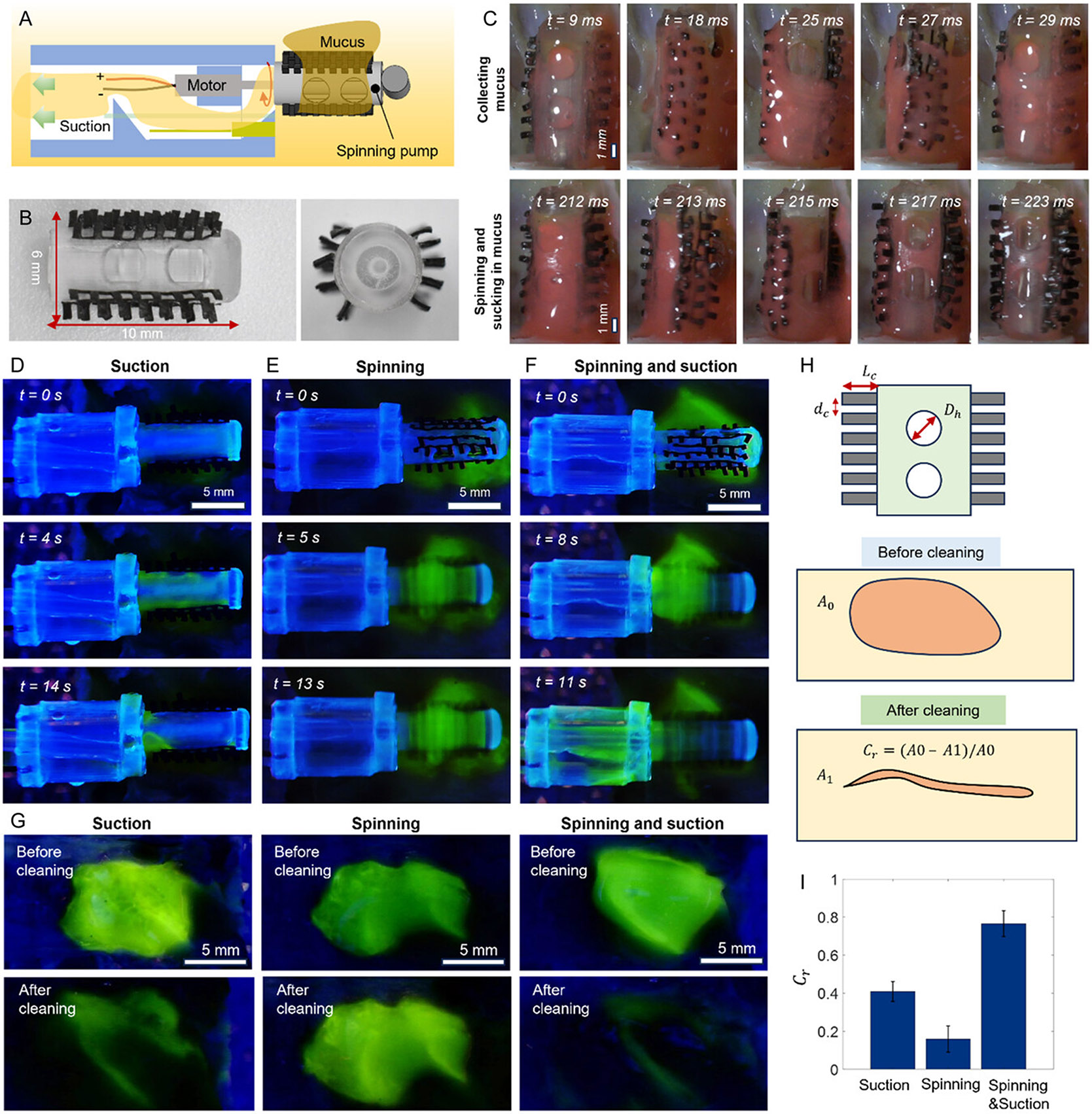
Mechanism of mucus removal via artificial cilia sweeping and suction. A) Schematic illustration of the mucus-cleaning mechanism combining mechanical sweeping and suction. B) Images of the ciliary cleaning head in the side view and bottom view. C) High-speed video (movie S1, Supporting Information) frames showing the combined action of mechanical cilia sweeping and suction. Mucus composition: mucus to water ratio by weight is 1:5. D–F) Video (movie S1, Supporting Information) frames of mucus removal using (D) suction, (E) mechanical sweeping (spinning) alone, and (F) both suction and mechanical sweeping (spinning). Liquid composition: 0.1 mL glycerol for ease of visualization. Fluorescence dye (excitation wavelength: 365 nm) is mixed in the liquid, and the images are taken in an ultraviolet (UV) chamber. G) Optical images of tracheal tissue before and after being cleaned with suction alone, mechanical sweeping alone, and combined suction and mechanical sweeping. H) Schematic illustration of the spinning pump design and the mucus cleaning ratio, defined as $C_{r}=1-A_{1}∕A_{0}$, where $A_{0}$ and $A_{1}$ represent the initial and post-cleaning mucus-covered areas, respectively. $L_{c}$ is the length of the artificial cilia. $D_{h}$ is the diameter of the hole on the cleaning head, while $d_{c}$ is the spacing of the artificial cilia. I) Mucus cleaning ratios using three methods demonstrated in (G). Error bars represent standard deviations for *n* = 3 trials.

The fundamental cleaning mechanism relies on mechanical artificial cilia and distributed suction. As illustrated in Figure 2A, the artificial cilia sweep across the tissue surface, mechanically collecting the mucus and guiding it toward the inlets. The viscous mucus gradually accumulates at the inlets, where negative pressure generated by the suction tube draws it into the cleaning head. It is then pumped away from the tissue surface. Five arrays of artificial cilia are made of composite of elastomer and magnetic particles, each measuring 1 mm in length, 0.6 mm in width, and 0.25 mm in thickness. They are integrated into the cleaning head by adhesive-based bonding as shown in Figure 2B. Figure 2C captures the cleaning process: the initial mechanical wrapping is visible in the high-speed video before *t* = 29 ms, followed by rapid suction of the mucus into the cleaning head between *t* = 212 ms and *t* = 223 ms, during which the mucus is effectively removed.

To investigate the synergistic effects of mechanical sweeping and suction, we conducted separate experiments under three conditions: suction only, mechanical sweeping only via soft ciliary brush, and the combined action of both. Figure 2D first presents the mucus clearing experiment using a cleaning head configured with the same artificial cilia distribution but equipped with two 2.5 mm diameter inlet holes for suction. The sequential images show that suction alone can remove nearby mucus; however, as the mucus layer thins, its removal becomes less effective. This inefficiency arises because gaps between the inlet, and the tissue surface allow air to enter, reducing the negative pressure and impairing mucus extraction. Figure 2E then shows the performance of mucus clearing by mechanical sweeping using the same cleaning head without activating the suction. While the artificial cilia effectively wrap and gather mucus, the accumulated mucus remains on the spinning pump surface, which has limited capacity for further collection and clearance. In contrast, Figure 2F demonstrates the combined operation of mechanical sweeping and suction. This approach efficiently attracts mucus to the head, even as the mucus layer becomes progressively thinner. The suction then transports the mucus through the inner suction tube. Within 24 s, the tissue surface is thoroughly cleared of mucus.

Furthermore, as shown in Figure 2G, fluorescence images comparing tissue surfaces before and after cleaning under each condition reveal that the combined method leaves the least residual mucus, validating the enhanced cleaning efficiency achieved by integrating mechanical sweeping and suction. To quantify cleaning efficacy, in Figure 2H, we defined a metric called the cleaning ratio, denoted as $C_{r}=1-A_{1}∕A_{0}$, where $A_{1}$ and $A_{0}$ represent the tissue areas covered by mucus before and after cleaning, respectively. A higher $C_{r}$ indicates more effective mucus removal. Figure 2I confirms that with both suction and ciliary sweeping, the mucus cleaning ratio is the largest which is twice that by only suction and four times of that by only sweeping, demonstrating the advantage of the proposed cleaning mechanism.

Finally, we comprehensively characterized the mucus materials used in our experiments (Figure S5, Supporting Information). The viscosity–shear rate profiles were measured to match the physiological range of CF and COPD mucus (8–10 Pa·s at low shear rates).^[5]^ Mucus were prepared using defined mucin–water mixtures, with viscosity and component ratios recorded for each formulation. Mucus cleaning performance was evaluated by measuring the flow–vacuum relationship and determining operating setpoints. To compare our device with a commercial therapeutic bronchoscope with only suction (Figure S6, Supporting Information), we ensure fair comparison and reproducibility by allowing similar flow curves and evaluating the cleaning performance in terms of volume rate.

**Figure 3.**
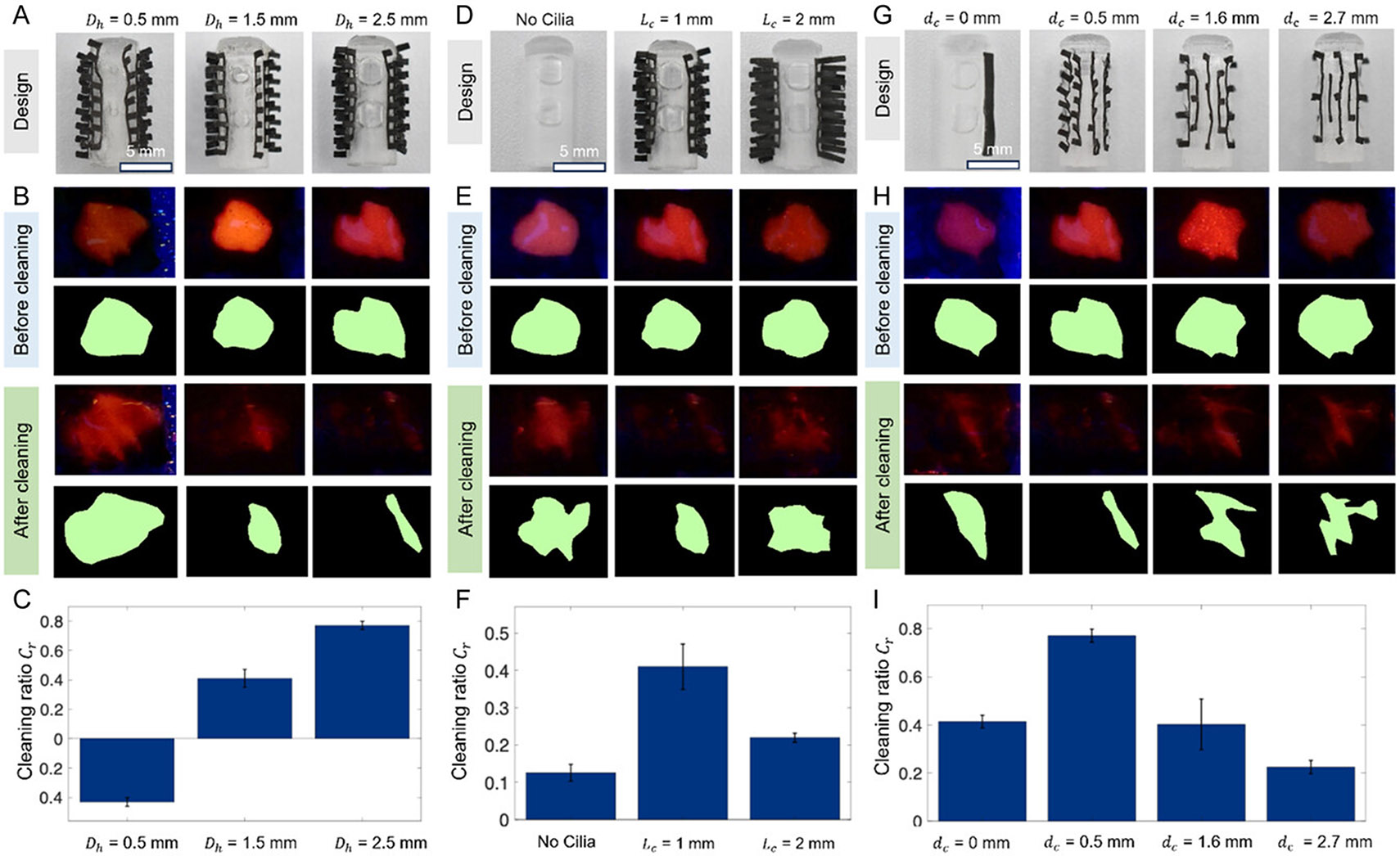
Characterization of the mucus cleaning tool with varying design parameters. A) Optical image of the device and segmented trachea images showing residual mucus after cleaning with spinners of different inlet diameters (0.5, 1.5, and 2.5 mm). B) Fluorescence images of the tissue surface with mucus before and after cleaning for the three designs in (A). C) Quantified cleaning ratios corresponding to the experiments in (B). D) Optical image of the device and segmented trachea images for spinners with artificial cilia lengths of 0.0, 1.0, and 2.0 mm. E) Fluorescence images of the tissue surface with mucus before and after cleaning for the three designs in (D). F) Cleaning ratios corresponding to the experiments in (E). G) Optical image of the device and segmented trachea images for spinners with artificial cilia spacing of 0.5, 1.6, and 2.7 mm. H) Fluorescence images of the tissue surface with mucus before and after cleaning for the three designs in (G). I) Cleaning ratios corresponding to the experiments in (H). Error bars indicate standard deviations for *n* = 3 trials.

To optimize mucus cleaning performance, we systematically investigated three key design parameters that govern the combined action of ciliary sweeping and suction pumping in Figure 3 and movie S2, Supporting Information. The key design parameters include the suction inlet diameter, artificial cilia length, and artificial cilia spacing. These parameters were chosen because they directly determine the catheter’s ability to mechanically gather mucus and then transport it away through fluid evacuation. We first examined the effect of suction inlet diameter ($D_{h}=0.5$, 1.5, and 2.5 mm) as shown in Figure 3A. In each experiment, 2 mL of porcine mucus mixed with fluorescent microparticles (30 μm, polyurethane, Cospheric Inc.) was applied to freshly harvested ovine tracheal tissue. Each cleaning head design was tested in triplicate under identical conditions. Fluorescence images before and after cleaning (Figure 3B) were segmented to quantify mucus coverage, and the cleaning ratios are summarized in Figure 3C. At the smallest diameter (0.5 mm), the viscous mucus was too thick to be effectively aspirated, often spreading along the tissue surface rather than entering the inlet. With an intermediate size of 1.5 mm, mucus removal improved significantly, and at 2.5 mm, the cleaning ratio reached ≈0.8, indicating efficient clearance. While further increases in inlet size could enhance performance, they are limited by the physical dimensions of the cleaning head and the need to maintain structural integrity.

Next, we studied the influence of artificial cilia length ($L_{c}=0.1$, 1, 2 mm) using cleaning heads with six ciliary arrays (Figure 3D). Without any artificial cilia $\left(L_{c}=0\;\text{mm}\right)$, the device relied solely on suction, which proved insufficient as mucus tended to spread across the tissue surface instead of being mobilized. Introducing medium-length cilia $\left(L_{c}=1\;\text{mm}\right)$ enabled effective wrapping and sweeping of mucus around the spinning pump, greatly facilitating its transport toward the inlets. This length offered the best balance between effective sweeping and minimal resistance. When the artificial cilia length was increased to 2 mm, however, the elongated structures required compression to maintain surface contact. This excessive contact increased friction, often stalling the motor and deforming the artificial cilia, which reduced their ability to gather and transport mucus. Quantitative analysis (Figure 3E,F) confirmed that the 1 mm length provided the most efficient cleaning.

Finally, we investigated the role of artificial cilia spacing ($d_{c}=0$, 0.5, 1.6, 2.7 mm) in mucus clearing as shown in Figure 3G. Closely packed artificial cilia (0 mm spacing) left little room for mucus to flow, reducing the sweeping effect. In contrast, widely spaced artificial cilia (2.7 mm) failed to adequately engage with the mucus layer, leaving large regions uncleared. An intermediate spacing of 0.5 mm provided the optimal balance by ensuring sufficient surface engagement while maintaining flow pathways for mucus transport. Results in Figure 3H, I confirm that this configuration yielded the highest cleaning ratio. Overall, these systematic investigations reveal the critical importance of tuning the inlet hole size and artificial cilia architecture to achieve efficient mucus clearance. The findings show that a 2.5 mm inlet hole, 1 mm artificial cilia length, and 0.5 mm artificial cilia spacing represent a promising set of design parameters, maximizing the synergistic effects of mechanical sweeping and suction pumping.

### 2.3. Mechanism of Sensing Touch on Biological Tissues by Measuring the Rotating Speeds

Effective mucus cleaning requires not only strong clearance capability but also careful control of the interaction between the spinner head and the airway tissue surface. Excessive contact pressure may damage fragile airway tissue or stall the motor, while insufficient proximity prevents the cleaning head from engaging mucus effectively. Thus, continuous monitoring of spinner–tissue interaction is challenging yet critical for balancing safety and efficacy.

**Figure 4.**
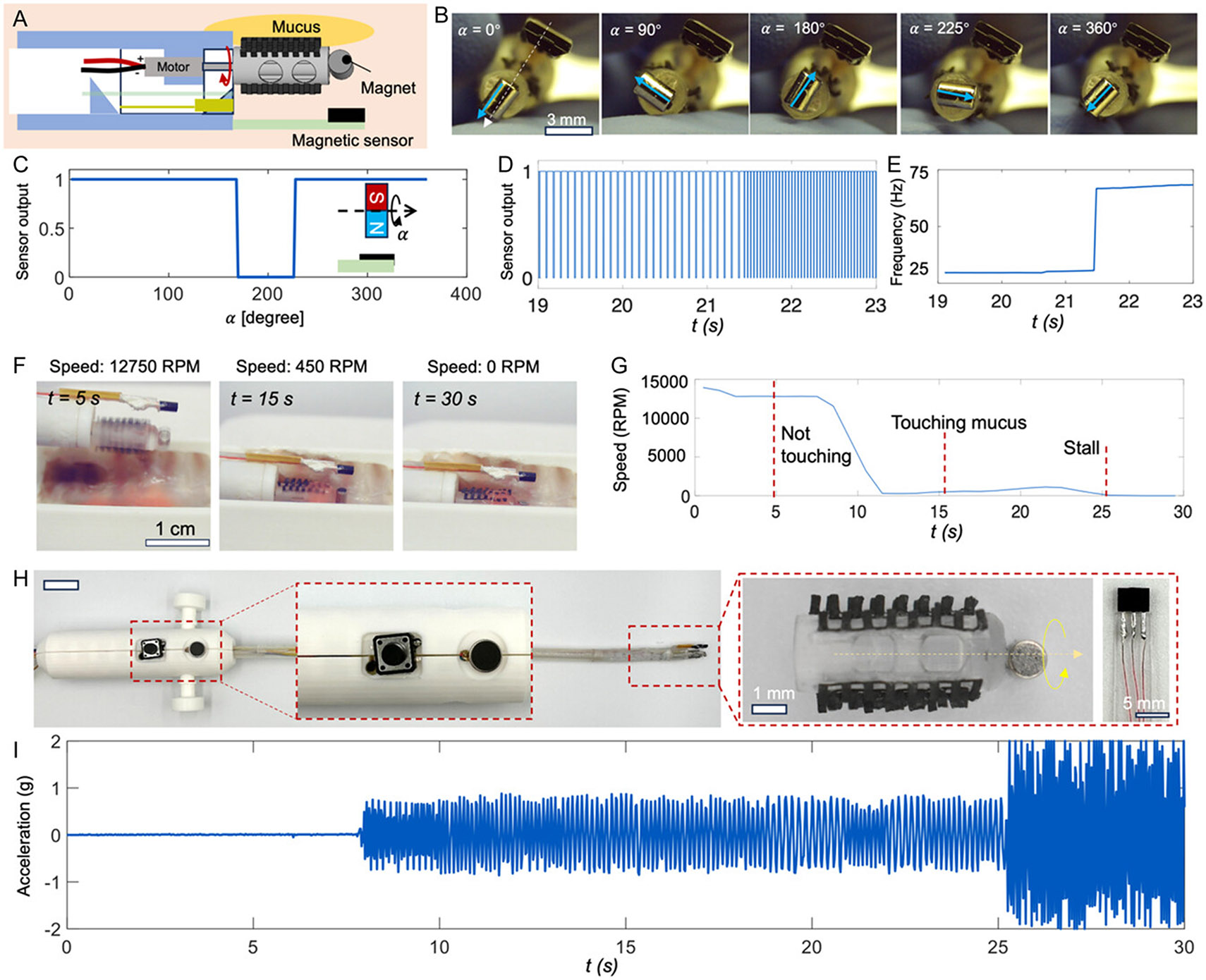
Mechanism for sensing spinner speed using a magnetic sensor and tracker. A) Schematic illustration of the sensing mechanism for spinner speed detection based on magnetic tracking. B) Representative video (movie S3, Supporting Information) frames showing the sensor signal as the magnetic marker passes over the sensor at various rotation angles. C) Sensor output when the spinner is rotating in a full period. D) Real-time magnetic sensor output as a function of time, demonstrating signal variation with different rotating frequencies. E) Extracted spinner frequency data corresponding to the measurements shown in (D). F) Video (movie S3, Supporting Information) frames capturing changes in the spinner-to-tissue surface distance during operation. G) Time-dependent measurement of the spinner speed corresponding to the distance variation process in (F). H) Optical image of the sensor and haptics integrated catheter with the Hall-effect magnetic sensor and the attached magnetic marker used for speed sensing. I) Measured acceleration output from the vibration motor as the spinner-to-tissue distance is modulated.

To address this challenge, we developed a real-time sensing approach based on magnetic tracking of spinner motion. A cylindrical permanent magnet (OD, 1.5 mm; length, 2.5 mm) was embedded into the spinner head as a magnetic marker (Figure 4A). A magnetic sensor board was mounted near the catheter tip, forming a compact and robust configuration for detecting local magnetic field variations. As the spinner rotates, the magnetic field oscillates periodically, which can be continuously recorded by the sensor as shown in the optical image of the setup in Figure 4B. Figure 4C illustrates the signal output corresponding to sequential images of one full spinner rotation. The critical signal changing angle is about 170 degrees and 225 degrees due to the distribution of the magnetic field generated by the cylindrical magnet in the direction of the sensor sensing axis. Figure S7, Supporting Information, further shows the magnetic field distribution using a discretized magnetic dipole model (see “Experimental Section”). With this sensing ability, Figure 4D demonstrates that changes in the spinner speed are directly reflected in the frequency of the recorded waveform. By analyzing the signal period, the rotational frequency of the spinner can be quantified accurately in real time, as validated in Figure 4E. This sensing method thus provides a simple and reliable proxy for monitoring spinner dynamics during operation.

We next investigated how this sensing mechanism can function as a proximity indicator. As the spinner approaches the tissue surface, increasing viscous and frictional drag gradually slows its rotation. This behavior is captured in Figure 4F, where the spinner speed progressively decreases as contact is approached, ultimately reaching near-zero when full contact is made. Quantitative results in Figure 4G reveal a spinner speed estimation consistent with the observation in Figure 4F, demonstrating that rotational frequency can serve as a sensitive indicator of proximity. Importantly, this strategy avoids the need for direct force sensors, which would complicate the design, and instead leverages the inherent motor–tissue interaction as a natural feedback signal.

To translate this sensing capability into feedback information for the operator, we implemented a haptic feedback system integrated into the catheter handle. As shown in Figure 4H, a miniature vibration motor was embedded in the handle and interfaced with the magnetic sensor via a motor driver all controlled by an electronic board (Figure S8, Supporting Information). The control scheme is straightforward: Spinner speed data are continuously converted into the vibration intensity. When the spinner rotates freely away from the biological tissue, the vibration is minimal. As the spinner is close to the airway surface and slows down, the vibration amplitude and frequency increase proportionally, providing an intuitive tactile cue of proximity. Figure 4I confirms this feedback mechanism experimentally, where inertia measurement unit (IMU) measurements quantify the increasing vibration strength as spinner speed decreases. This integrated haptic sensing system transforms a potentially unsafe, visually demanding task into an intuitive, operator-friendly procedure. The clinician can sense in real time whether the cleaning head is at a safe working distance, reducing the risk of tissue damage while ensuring effective mucus clearance. By embedding magnetic tracking and haptic feedback into the catheter system, we provide a closed-loop interface that not only enhances operational safety but also lays the foundation for future autonomous or semi-autonomous airway-cleaning devices. Finally, we performed force calibration for the haptic feedback system (Figure S9, Supporting Information), and a safety threshold can be incorporated based on the calibrated force range. In future work, we plan to integrate pressure sensors on the device for real-time monitoring of tissue contact force. As shown in Figure S10, Supporting Information, no visible tissue damage was observed during or after the brushing experiments, confirming the safety of our device under the tested conditions.

### 2.4. Demonstration of Catheter Steering, Camera Guided Navigation, and Targeted Mucus Cleaning

**Figure 5.**
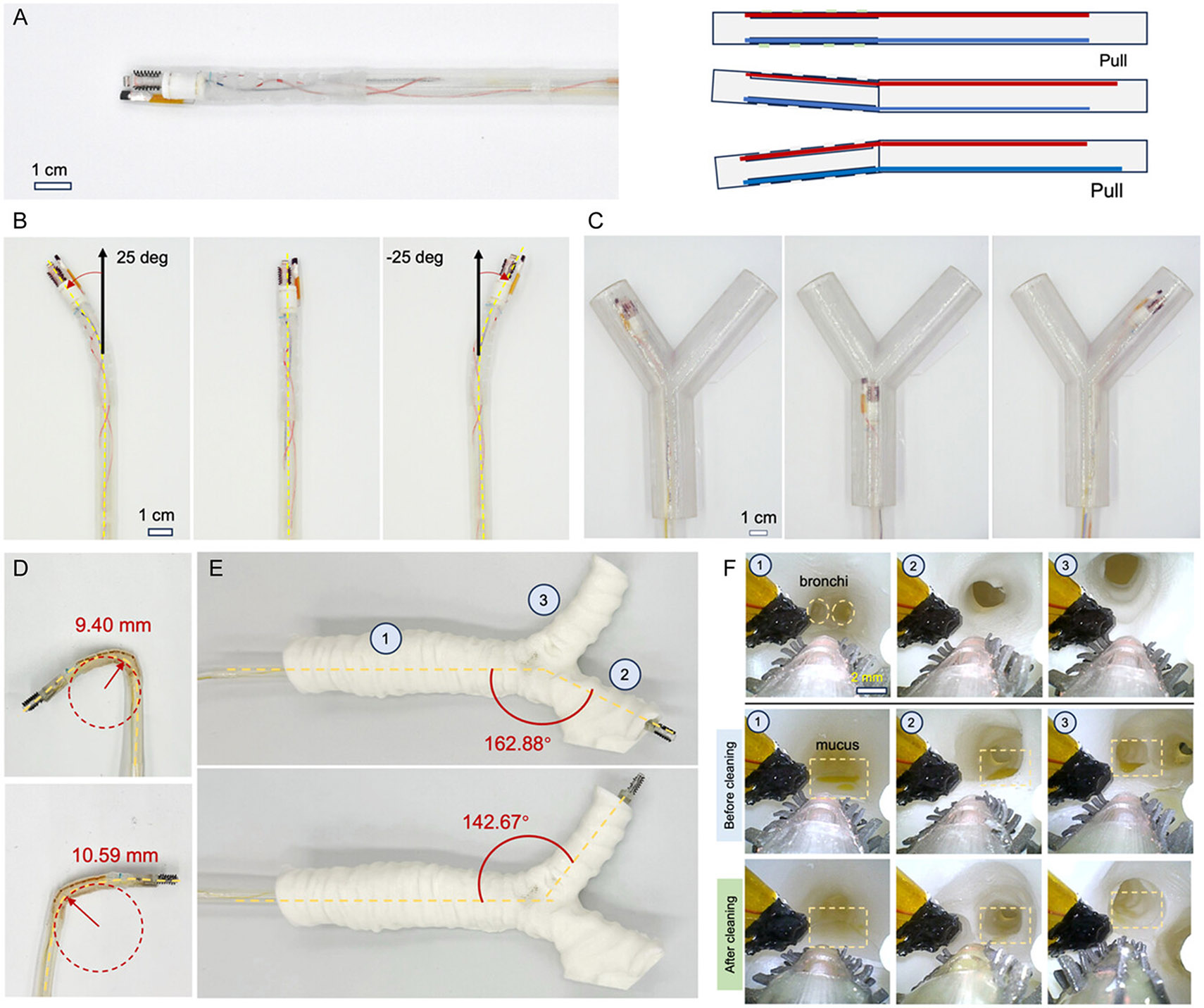
Steering control and functionality of the mucus-cleaning tool within a phantom model. A) Schematic illustration of the tendon-driven steering mechanism. There are notch-like structures on the steering tube which are bonded with two nylon strings for steering. B) Demonstration of steering the catheter at various deflection angles by pulling the two strings accordingly. C) Sequential optical images showing the catheter navigating inside a “Y”-shaped phantom. D) Minimal radius achieved by the steerable catheter without collapsing. E) Steering angle inside a 3D-printed phantom of trachea. F) On-board camera view inside a trachea phantom during the navigation. Three locations are marked on the phantom. Images of the three locations are shown before and after the mucus are cleaned.

Since this work focuses on the cleaning mechanism, we implemented a simple tendon-driven steerable sheath. The approach of mucus cleaning, however, is compatible with other steering strategies as needed. To improve maneuverability for effective mucus clearance in the airway, we developed a steerable catheter equipped with a sheath actuated by two embedded cables. The sheath incorporates strategically placed notches near the cleaning head, as illustrated in Figure 5A, to facilitate bending. Two nylon strings are anchored to the front tip of the notched structure, enabling directional control: when one string is pulled, the structure bends toward that side, thereby steering the catheter. This simple yet robust mechanism allows ±25° bending in both directions (Figure 5B), providing sufficient flexibility to navigate the confined and branching geometries of the trachea and bronchi.

We next validated the catheter’s maneuverability and mucus-cleaning capability in a phantom model designed with a Y-shaped tubular structure (Figure 5C). A miniature camera (OD: 2 mm) was integrated at the catheter tip, enabling real-time visualization of the lumen to guide steering and ensure precise navigation of the cleaning head. The steerable sheath can achieve a minimal bending radius of 9.5 mm as shown in Figure 5D. Figure 5E further shows an airway phantom for human adults (average trachea diameter: 18 mm). The catheter is controlled to navigate through the trachea and bronchi for mucus cleaning. By coordinating visual feedback with cable actuation, the catheter successfully traversed branching airways and reached designated sites within the phantom. Figure 5F and movie S4, Supporting Information present representative camera images acquired during the steering process as the catheter navigated through the phantom and performed mucus removal tasks. Three locations within the phantom are marked and imaged both before and after the cleaning procedure. The comparison of these images clearly demonstrates a reduction in mucus coverage ratios, confirming the effectiveness of the steerable sheath and cleaning head in clearing airway obstructions. Overall, this steerable catheter system combines directional control, real-time visualization, and cleaning capability, providing a versatile platform for addressing mucus accumulation in the trachea and bronchi. Its ability to achieve controlled navigation and effective clearance highlights its potential for translation to clinical airway management.

**Figure 6.**
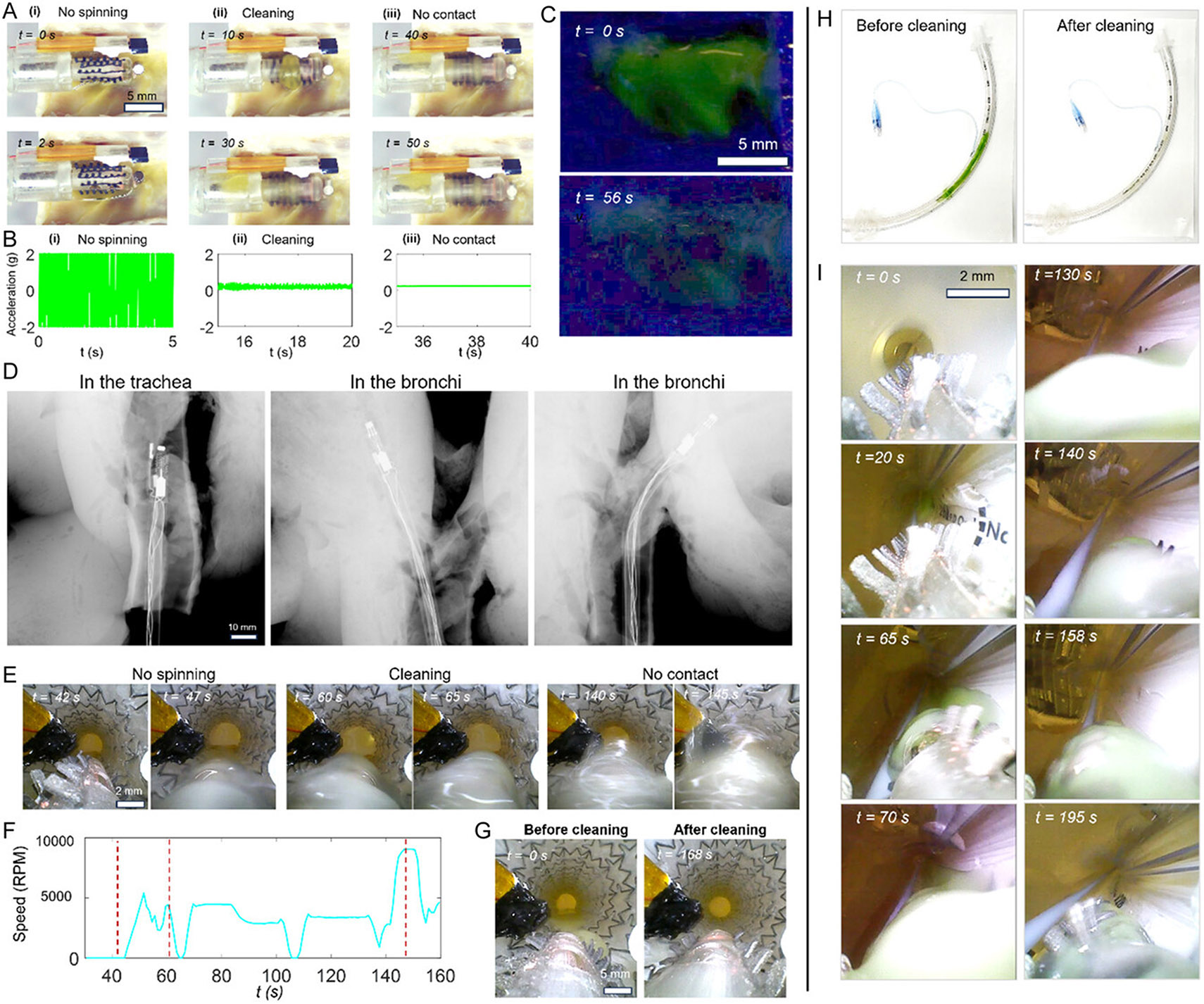
Demonstration of mucus cleaning in a phantom model and ex vivo ovine trachea. A) Video (movie S4, Supporting Information) frames of ex vivo mucus cleaning on ovine tracheal tissue. i) No spinning with full contact. ii) Cleaning. iii) No contact. B) The corresponding haptic feedback signals: i) spinner off, ii) active cleaning at low spinner speed, and iii) no contact between the cleaning head and tissue. C) Optical images of tracheal tissue before and after cleaning. D) X-ray images of the catheter positioned inside an ovine lung. E) Video (movie S4, Supporting Information) frames of mucus removal from within an airway stent. F) Measured spinner speed as a function of time during the cleaning process shown in (E). G) Optical images of the airway stent before and after cleaning. H) Optical images of an endotracheal tube with an inner diameter of 9.0 mm before and after being cleaned. I) Video (movie S4, Supporting Information) frames of the cleaning process inside an endotracheal tube recorded using an on-board camera.

To show the ability of targeted mucus clearance, we demonstrated mucus cleaning inside an ex vivo ovine trachea, airway stent and airway endotracheal tubes, using the developed catheter. In the first demonstration, we validated the mucus cleaning performance in an ex vivo ovine trachea using the cleaning head integrated with a haptic sensing interface. A miniature camera assists in guiding the catheter, while the catheter’s steering capability enables precise adjustment of the cleaning head’s distance from the tissue surface. As shown in Figure 6A, real-time video frames captured by the on-board camera illustrate various stages of the cleaning process: initial contact with the tissue where the spinner is stalled, active cleaning as the head is steered slightly away from the tissue, and full detachment where the head loses contact with the surface. Figure 6B presents the corresponding sensor signals recorded by the magnetic tracking system, offering continuous feedback on the spinner’s motion and its interaction with the biological tissue. Figure 6C shows the fluorescence images of the tissue surface before and after cleaning using the sensor-integrated catheter, which combines both mechanical cilia-based sweeping and suction. The results demonstrate effective mucus removal with minimal tissue damage, highlighting the benefit of real-time feedback in optimizing cleaning performance while ensuring tissue safety. Finally, the device could be clearly visualized using computed tomography to help observe the device status as shown in Figure 6D.

As an additional demonstration, we performed mucus cleaning inside an airway stent, which is highly susceptible to plugging due to mucus accumulation and obstruction by airway cilia. Airway stents are covered by a polymer mesh to avoid tissue ingrowth, but they also block the biological cilia and impair mucociliary clearance. Therefore, excessive mucus accumulation often happens in an airway stent which causes inflammation and difficulty of breathing. Conventional blind suction methods pose a considerable risk of airway damage as well as stent migration. In contrast, our system enables controlled mucus removal with integrated haptic feedback (Figure 6E). The haptic unit produces vibrations whose amplitude and frequency correspond to variations in spinner speed. Within one minute, the mucus was completely removed without causing any mechanical displacement of the stent. The recorded spinner speed during cleaning is shown in Figure 6F, confirming stable operation. Figure 6G further compares the stent before and after cleaning, demonstrating that patency was fully restored without noticeable migration.

Finally, we also tested a catheter (OD: 6 mm) incorporating the same cleaning mechanism for use in an endotracheal tube with an inner diameter of 9.0 mm (Figure 6H). In this design, the miniature motor was replaced with a flexible shaft (nitinol wire, OD: 0.3 mm), while all other components were retained, reducing the catheter diameter to 6 mm. As shown in Figure 6I, the onboard camera records both navigation and mucus removal, confirming the catheter’s effectiveness for cleaning mucus in another airway device. This scaled down design also enables navigating smaller airway structures (down to 7 mm) in a phantom as shown in Figure S11, Supporting Information.

## 3. Conclusion and Discussion

We have developed and demonstrated a steerable catheter with integrated mucus removal functionality for use during bronchoscopy procedures. The device combines soft artificial cilia with suction for active mucus clearance and incorporates a sensing unit to detect contact and a vibration motor for providing haptic feedback. A tendon-driven steerable sheath enables precise navigation into airway branches. With a compact diameter of ≈8 mm, the system can access both the trachea and bronchi, performing on-demand mucus removal under real-time visualization via an onboard camera. This targeted cleaning capability could potentially facilitate the localized delivery of therapeutic agents, including drug patches and stents. We have systematically evaluated the device’s performance of mucus cleaning in phantom models, ex vivo ovine lungs, and airway prosthetic devices. The catheter has been demonstrated to avoid damage of surrounding tissues when cleaning mucus directly on airway tissues and preventing stent migration when cleaning mucus plugged airway stents. Compared with therapeutic bronchoscopes, the catheter provides faster and more effective clearance, addressing critical needs in intensive care patients where bronchoscopy duration is constrained by ventilation challenges. This technology offers a promising strategy for safe, efficient, and timely airway mucus management.

The current prototype has an outer diameter of 8 mm, which may limit its applicability in narrower airways. As a proof-of-concept, we demonstrated reducing the diameter of the cleaning head to be around 6 mm by using a flexible shaft,^[31]^ which could be further reduced by scaling down the spinning speed sensing unit and relocating its location as demonstrated in Figure S11, Supporting Information. The flexible-shaft-based miniature catheter could perform mucus cleaning in a trachea phantom model accessing smaller branches, validating the feasibility of the design. This demonstration highlights the potential for further miniaturization of the catheter system. In addition, flexible sensors may be used for tactile sensing which allows more sensitive touch sensing.^[32,33]^ Furthermore, advanced functions such as real-time mucus property sensing mechanisms including the sensors shown in our previous works on sensing mucus viscosity^[34,35]^ and layer thickness,^[35]^ tissue stiffness^[36]^ for detecting fibrosis or tumor, and integrated drug eluting^[37]^ or tissue biopsy^[38]^ functions could be further implemented on the catheter. Lastly, the haptics feedback interface could also be extended to touch sensing^[39]^ using a capacitive strain gauge with more sensitive pressure sensing mechanisms.^[40]^

Toward clinical application, the catheter may experience reduced performance when operating in highly viscous mucus or when the cleaning head is positioned too close to tissue. We empirically characterized motor torque, rotational speed, tip temperature, acoustic noise, irrigation flow, and suction pressure to ensure stable operation (Figure S12, Supporting Information). Potential failure modes, such as motor stall, jamming, or tissue entanglement, are detectable through magnetic sensor signal variations, enabling adaptive control. Integrated haptic feedback provides real-time tactile cues to the operator, facilitating early detection of abnormal conditions and ensuring safe manipulation.

In the future work, we plan to integrate advanced sensors and develop a deployment mechanism for drug patches following mucus clearance.^[41]^ After removing the mucus, targeted therapies such as gene delivery^[42]^ could be more efficient for treating underlying conditions. In addition, drug eluting stents^[43,44]^ which are tube structures with coated drugs, could be used after mucus cleaning to enable long-term drug release. We will also explore miniaturized pumping mechanisms or structural modifications to enable access to more confined airway regions, broadening the device’s clinical utility. Nonetheless, the proposed mucus cleaning mechanism offers a promising solution for efficient and safe mucus clearance in the airway and within airway prosthetic devices. The smart catheter demonstrated with haptics feedback for mucus cleaning combing soft body pumping and suction could enable safe and intelligent airway clearance to address clinical challenges in various airway diseases.

## 4. Experimental Section

### Preparing the Mucus Cleaning Head:

A cylindrical component (height: 10 mm, OD: 4 mm) was designed in SolidWorks (Dassault Systèmes, France). The design included a hollow channel (outer diameter, 3.5 mm; inner diameter: 2 mm) to allow mucus flow. On one side surface of the cylinder, two holes (diameter: 2.5 mm) were added. At the center, a 2 mm solid cylindrical section was retained for the motor shaft after drilling the internal channel. This process was mirrored on the opposite side, generating two symmetrical recesses. On the bottom surface, a 1 mm diameter hole was created concentrically and extruded to a depth of 5 mm to accommodate the motor shaft. The model was imported into PreForm software (Formlabs Inc., USA) and fabricated using a stereolithography printer (Form 4) with UV-curable resin (Clear V5). The printed part was first cleaned in a sealed beaker of isopropyl alcohol (IPA) and subjected to ultrasonic cleaning for 20 min. Residual IPA was then removed with compressed air, followed by UV curing at 35 °C for 10 min to complete the post-processing workflow.

### Preparing the Artificial Cilia:

To mold the thin sheet for preparing the artificial cilia structure, four layers of Polyethylene Terephthalate (PET) tapes (total thickness ≈0.25 mm) were applied along the edges of a standard glass slide. Magnetic composite mixture was prepared by mixing Ecoflex 00-30 (Smooth-On, USA) with NdFeB microparticles (average diameter, 5 μm; MQFP-15-7, Neo Magnequench) with a weight ratio of 1:1. The objective of including NdFeB particles is to tune the elasticity of the artificial cilia and also facilitate assembly of the cilia array on the cleaning head using magnetic alignment. The composite material exhibits a Young’s modulus of ≈144 kPa and a density of (2.00 ± 0.056) × 10^3^ kg m^−3^ based on a 1:1 mixing ratio between Ecoflex 00-30 and NdFeB microparticles. The mixture was poured onto the taped slide and scratched to be flat using a straight-edged blade. Curing was performed on a hot plate at 70 °C for 1 h. A single row of eight artificial cilia, each measuring 1 mm in length and 0.6 mm in width with an inter-cilium spacing of 0.5 mm, was sketched using SolidWorks 2025 (Dassault Systèmes, France). The sketch was exported as a Drawing Interchange Format (DXF) file for subsequent fabrication steps. With the cured composite, the artificial cilia shapes were precisely laser cut from the elastomeric sheet using an LPKF ProtoLaser U4 micromachining system (LPKF Laser & Electronics AG, Germany). The machined artificial cilia were magnetized using a pulsed magnetic field alignment machine (ASC IM-10-30, ASC Scientific) for ease of assembly on the surface of the spinning pump with the assistance of external magnetic fields. To facilitate upright assembly, individual cilium was temporarily positioned vertically by placing a disk magnet beneath a glass dish and assembling the artificial cilia under a stereomicroscope. Using precision tweezers, each artificial cilium was transferred to the cleaning head surface. A fine polymer filament tip dipped in cyanoacrylate adhesive (super glue) was used to apply a small amount of glue at the base of each cilium. After allowing sufficient time for adhesion, the process was repeated to assemble five rows of artificial cilia, resulting in full coverage of the cleaning head with artificial cilia. To ensure biocompatibility, the magnetic particles were coated with SiO_2_ and the artificial cilia surfaces were coated with a thin layer of PDMS (Figure S13, Supporting Information). In the experiments shown in Figure S14, Supporting Information, the soft ciliary brush was used to clean biological tissue and subsequently rinsed in phosphate-buffered saline (PBS) by spinning the cleaning head. No debris were observed in the microscope images. Moving forward, we plan to tune the artificial cilia stiffness either by adjusting the beam thickness or by incorporating alternative biocompatible materials, such as SiO_2_-coated NdFeB or SiO_2_ nanoparticles.^[45,46]^

### Preparing the Steerable Sheath:

An elastic sheath was designed in SolidWorks 2025 (Dassault Systèmes, France) with an inner diameter of 7.5 mm, an outer diameter of 8 mm, and a notch gap of 2 mm, depending on the application. The part was modeled using the 3D sketch and solid features and then exported as a binary-format STL file with maximum resolution settings. The STL file was imported into PreForm software and printed in a Form 4 3D printer (Formlabs Inc., USA) using Elastic 50 A. Following 3D printing, the part was carefully removed from the build platform and submerged in a beaker filled with isopropyl alcohol (IPA). The beaker was placed in an ultrasonic cleaner and processed using the full-wave mode for 20 min. The part was then transferred into fresh IPA for an additional 10 min of passive cleaning. Afterward, residual IPA was removed by blowing dry air from the lab bench. The sheath was subsequently immersed in water within a clean beaker to ensure full submersion during UV post-curing. The beaker was placed into a UV curing chamber (Form Cure or equivalent) and cured at 60 °C for 30 min. Upon completion, the cured part was taken out, and all support structures were carefully detached using a razor blade. To integrate the actuation string, a nylon string was passed through the sidewall near the head of the sheath using a fine suture needle. The remaining length of the thread was routed through the interior channel of the sheath and pulled out from the opposite end. A secure knot was tied at an appropriate location to prevent dislodgement during actuation by the tensile force applied to the nylon string.

### Preparing the Suction Tube and Pump:

To assemble the motorized spinner module, a small amount of cyanoacrylate adhesive (super glue) was first applied to the exterior surface of the DC motor casing. The motor was then inserted into a custom-designed funnel structure and held in place for ≈1 min to allow the adhesive to solidify, ensuring a stable mechanical fixation. A hole was created on the side wall of the funnel to allow passage of the motor wires. The wires were routed through this hole, and additional super glue was applied to seal the entry point, both to secure the wiring and to prevent potential fluid leakage. Next, the suction tube (inner diameter, 3.1 mm; outer diameter, 4.2 mm) was aligned and affixed to the funnel using super glue to create a continuous fluid path (Figure S15, Supporting Information). The assembled motor–funnel–tube structure was then inserted into a prefabricated elastic sheath such that the upper rim of the funnel was flush with the upper edge of the sheath. A hole with a diameter matching that of the motor shaft was created at the center of the spinner component. The spinner was aligned with the motor shaft and affixed using a small volume of super glue to ensure proper adhesion and torque transmission. The suction tube was subsequently connected to an external vacuum pump system, and the electrical motor terminals were connected to a DC power supply (maximum voltage and power: 24 V, 480 W). Finally, the open end of the suction tube was sealed with a funnel head using super glue to ensure leak-tight operation. For functional testing, the vacuum pump was activated to verify the suction and spinner performance.

### Preparation of ex vivo Trachea Sample:

An ex vivo ovine trachea was prepared by excising a ≈50 mm segment using surgical scissors. The tracheal segment was then longitudinally bisected, and one half was reserved for side-view video recording during the experimental procedures. As the halved tracheal tissue was unable to maintain its natural cylindrical curvature, a custom support fixture was designed using SolidWorks 2025 (Dassault Systèmes, France). The fixture consisted of a cylindrical holder measuring ≈18 mm in diameter and 60 mm in length, providing a stable platform for mounting the tracheal tissue. The part was fabricated using a desktop 3D printer (X1-Carbon Combo, Bambu Lab), and the bisected trachea tissue was gently draped over the holder to preserve the anatomical orientation and enable consistent imaging during the experiment.

### Fluorescent Mucus Preparation and Imaging for Cleaning Performance Assessment:

To assess cleaning performance, a fluorescently labeled mucus analog was prepared by mixing mucin from porcine stomach (Type II, Sigma Aldrich) in deionized water at a 1:5 weight ratio. A total of 0.15 mL of this mucus solution was drawn into a syringe and dispensed into a small weigh boat. Fluorescent polyethylene microspheres (diameter: 32–53 *μ*m, Cospheric LLC) were added to the solution to enable optical visualization. The mixture was thoroughly stirred to achieve a homogeneous suspension of microspheres within the mucus. The fluorescent mucus was then carefully applied onto the surface of the tissue sample, which was immediately placed inside a custom light-shielded imaging box equipped with three ultraviolet (UV) lamps for fluorescence excitation. An initial image (“before cleaning”) was captured using a webcam and YouCam software under fixed imaging parameters (brightness = 19, contras*t* = 100, exposure = −2). Under these conditions, only the fluorescent signal from the labeled mucus was visible against a dark background. Following initial imaging, the tissue was removed from the box, and a catheter-based cleaning procedure was performed. The tissue was then repositioned within the same imaging setup, and a second image (“after cleaning”) was acquired using identical camera and lighting settings to ensure consistency in image comparison. This protocol was repeated for three times to ensure reproducibility of results.

### Quantitative Image Analysis of Mucus Removal:

To quantify the efficacy of mucus removal, fluorescence images acquired before and after cleaning were analyzed using MATLAB 2025a (MathWorks, USA). Each image was imported into the software, and the k-means clustering algorithm was applied to segment fluorescent regions from the background. The segmentation algorithm identified clusters corresponding to high-intensity fluorescent pixels associated with the labeled mucus. For each image, the mucus coverage ratio was calculated as the ratio of fluorescent pixels to the total number of pixels in the region of interest. This yielded a dimensionless value between 0 and 1, representing the fractional reduction in mucus coverage due to the cleaning procedure. This analysis was performed for each trial to enable quantitative comparison of cleaning performance.

### Magnetic Sensor Integration and Rotational Speed Measurement:

To monitor the rotational speed of the cleaning head, a Hall-effect magnetic sensor (model A3144E) was employed. The sensor features three terminals: VCC, GND, and Signal. Electrical connections were established by soldering three insulated wires to the corresponding pins. The sensor was mounted onto a transparent acrylic board and mechanically stabilized using polyimide (PI) tapes to secure the wires and prevent disconnection due to mechanical strain. To provide moisture resistance and electrical insulation, a thin layer of liquid silicone-based sealer was applied over the sensor and the exposed solder joints, minimizing the risk of short circuits from mucus contamination during operation. A cylindrical neodymium magnet (1.5 mm in diameter, 2.5 mm in length) coated with UV curable resin (Figure S16, Supporting Information) was affixed flat to the upper surface of the cleaning head using adhesive. As the motor-driven cleaning head rotates, the south pole of the magnet periodically passes over the Hall-effect sensor. When aligned, the sensor outputs a digital high signal (logic “1”); otherwise, it outputs a low signal (logic “0”), generating a square-wave output. The digital signal from the sensor was read using a LabVIEW data acquisition system (National Instruments). The number of rising edges (transitions from 0 to 1) was counted over a one-second interval, corresponding to the number of full revolutions per second. The rotational speed was then converted to revolutions per minute (RPM) for further analysis.

### Magnetic Field-Based Haptic Sensing:

The magnetic fields generated by a magnetic marker were modeled using a discretized magnetic dipole model.

(1) $$B(r)=\sum_{i=1}^{n_{1}}B_{m,i}(r;r_{i},m_{i})$$

r is the 3D position of the magnetic sensor in the space. $B_{m,i}(r;r_{i},m_{i})$ is the magnetic field generated by the i–th $i=1,\ldots ,n_{1}$ magnetic element on the magnetic marker.


(2) $$B_{m,i}=\frac{\mu_{0}}{4\pi }\left[\frac{3(r-r_{i})(m\cdot (r-r_{i}))}{{|r-r_{i}|}^{5}}-\frac{m_{i}}{{|r-r_{i}|}^{3}}\right]$$

### Statistics Analysis:

Data presentation (mean and standard deviations) and sample size (n) for each statistical analysis were mentioned in the figure caption. MATLAB 2025a (MathWorks Inc.) was used for statistical analysis.

## Supplementary Material

movie S1

movie S2

movie S3

movie S4

SI

Supporting Information is available from the Wiley Online Library or from the author.

## Data Availability

The data that support the findings of this study are available in the supplementary material of this article.
