# Supplementary material for: Soft Artificial Ciliary Brush with Integrated Haptic Feedback for Efficient Airway Mucus Cleaning
Source: Adv Intell Syst. Author manuscript; Available in PMC 2026 May 16. (PMC13178504; doi:10.1002/aisy.202501236)
Supplement: SI [file NIHMS2166332-supplement-SI.pdf]

Supplementary Information for

Soft Artificial Ciliary Brush with Integrated Haptic Feedback for Efficient Airway Mucus Cleaning

Zhongming Lyu[1], Yusheng Wang[1], Ruijian Ge[1], Darren Wang[1], Matthew Bacchetta[4], Caitlin T Demarest[5], Fabien Maldonado[1,4], Xiaoguang Dong[1,2,3,*]

[1] Department of Mechanical Engineering, Vanderbilt University, TN 37212, US
[2] Department of Biomedical Engineering, Vanderbilt University, TN 37212, US
[3] Vanderbilt Institute for Surgery and Engineering, Vanderbilt University, TN 37212, US
[4] Division of Allergy, Pulmonary and Critical Care Medicine, School of Medicine, Vanderbilt University, Nashville, TN 37232, US
[5] Department of Thoracic Surgery, Vanderbilt University Medical Center, Nashville, TN 37232, US

* Corresponding to xiaoguang.dong@vanderbilt.edu

The PDF file includes:

Fig. S1 to S16
Supplementary Note 1
Notes for movies S1 to S4

Other Supplementary Material for this manuscript includes the following:

movies S1 to S4

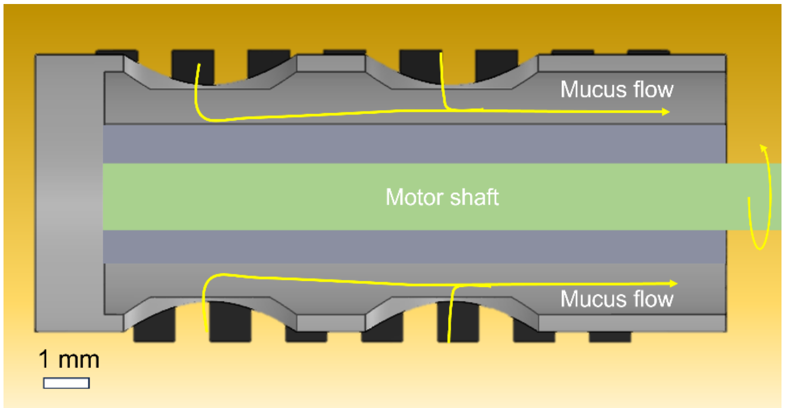

Fig. S1. 3D rendering of the spinning pump and soft ciliary brush. The spinning pump contains an internal hollow channel for insertion of a motor shaft or other transmission shaft. Mucus enters through surface openings on the spinning pump and is directed into the hollow channel, where it is transported into the suction tube for removal.

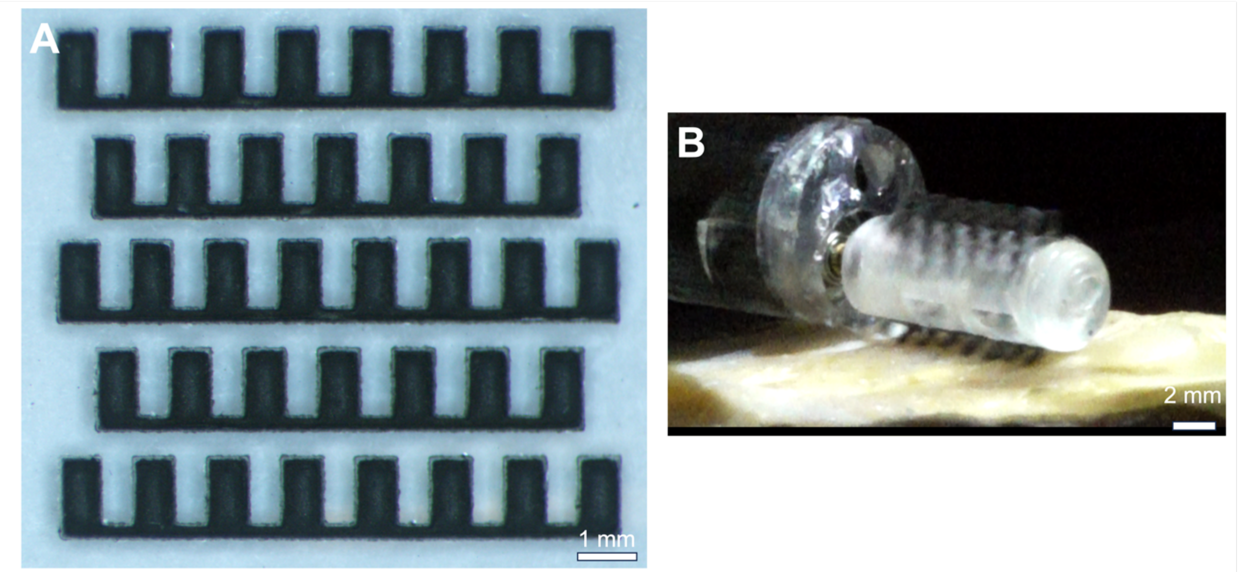

Fig. S2. Optical images of artificial cilia arrays and their deformation on tissue surfaces. (A) Artificial cilia arrays fabricated by mixing Ecoflex 00-30 with NdFeB microparticles, followed by laser machining to achieve the desired shapes and dimensions. (B) Deformation of artificial cilia on ovine tracheal tissue, demonstrating their softness and safe interaction with biological surfaces.

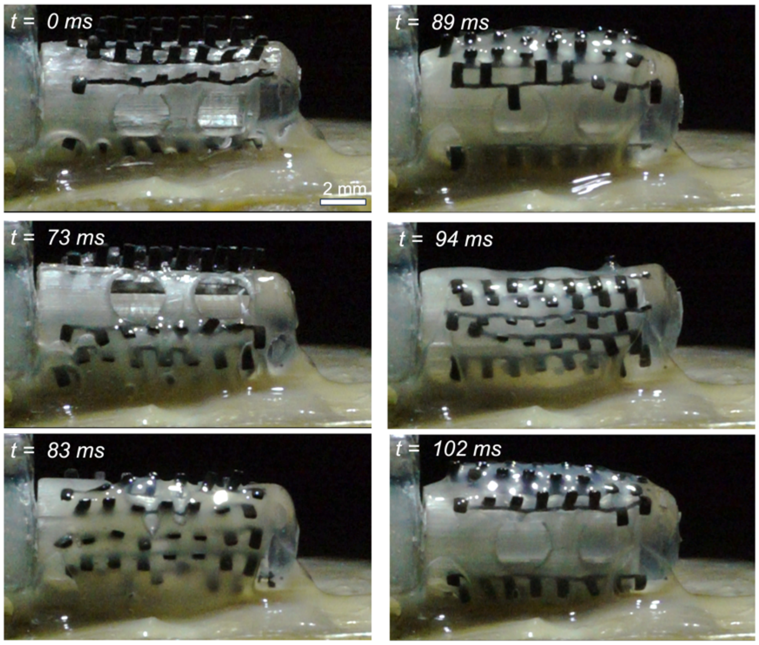

Fig. S3. High-speed video frames showing the mucus scratching process. The mucus was prepared by mixing porcine stomach mucin with water at a weight ratio of 1:5 and applied onto ovine tracheal tissue.

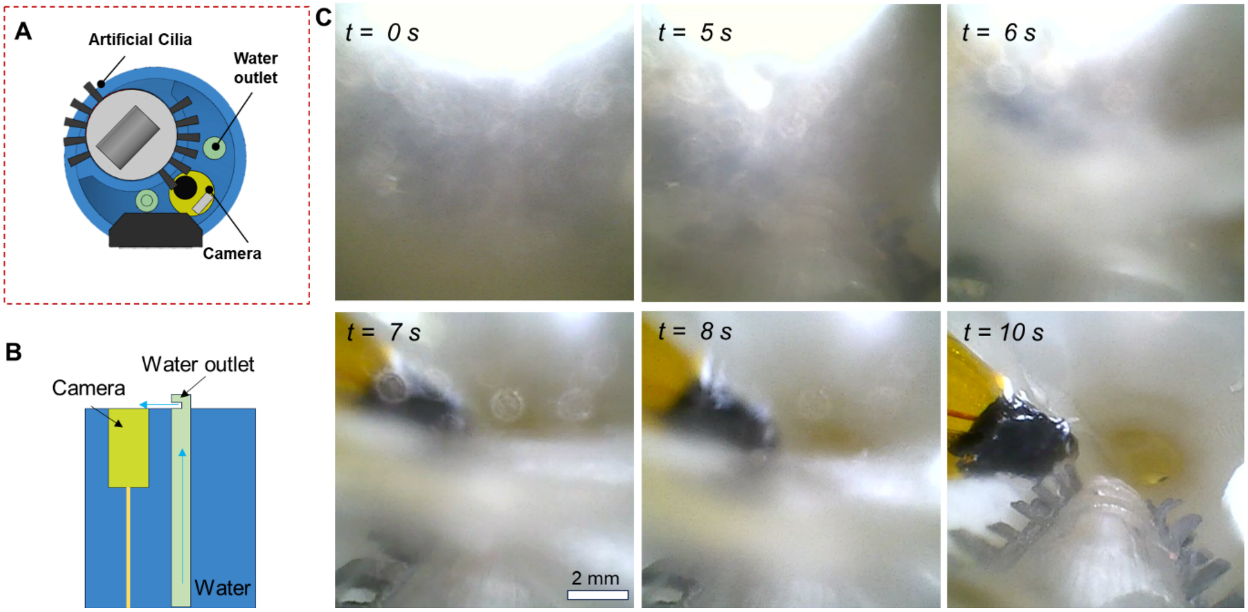

Fig. S4. Demonstration of camera lens cleaning with water flushing. (A) Illustration of water outlets designed for lens flushing. (B) Side view of the water channel. (C) Video frames showing removal of mucus contamination from the camera lens by water flushing. The outlet is positioned on the side of the tube, directing flow onto the lens surface.

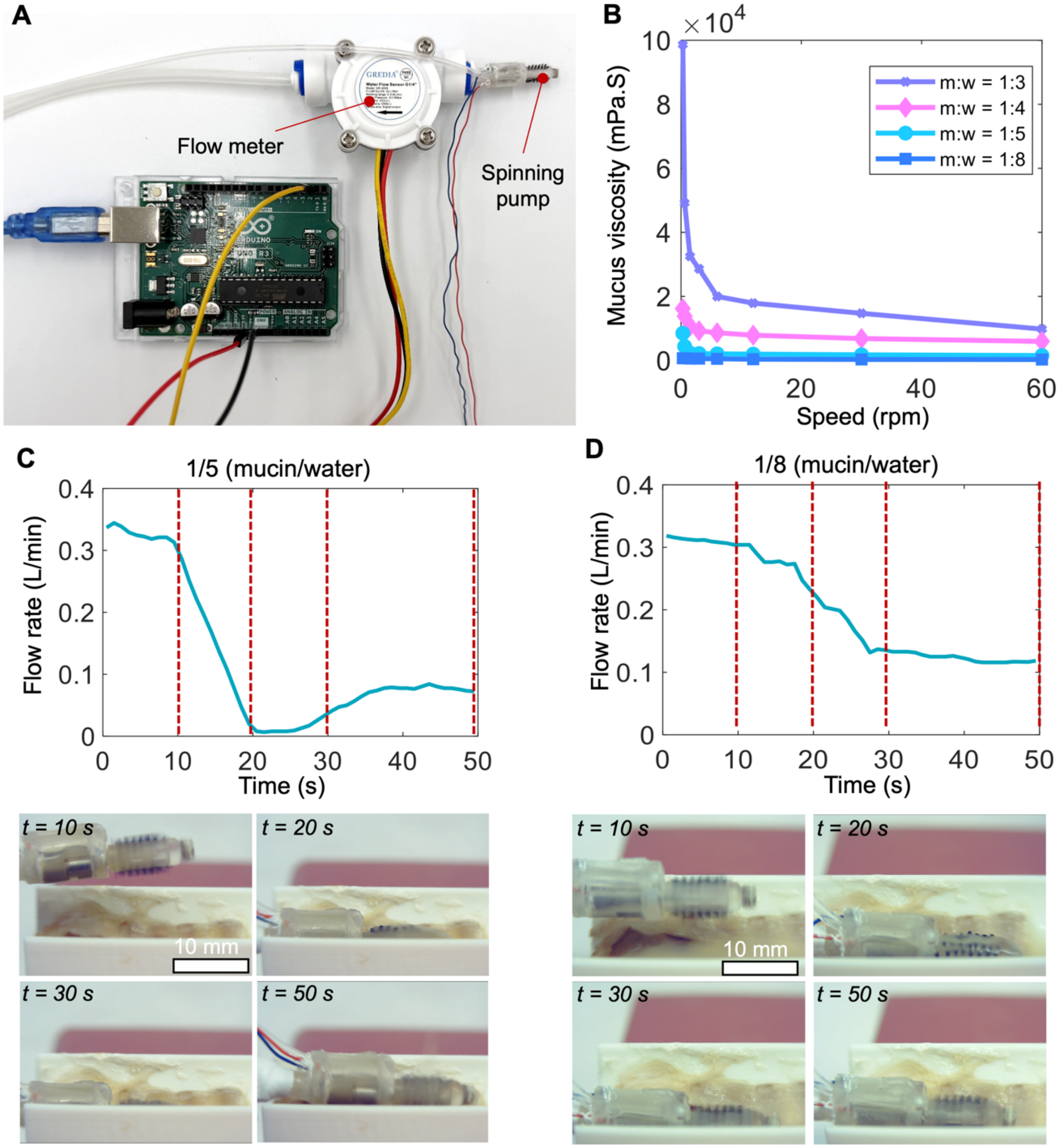

Fig. S5. Characterization of suction flow and mucus viscosity. A. Experimental setup with an integrated flow sensor for flow rate measurement. B. Measured viscosity of the mucus samples used in the experiments. C. Suction flow rate when using mucus with a water-to-mucin mixing ratio of 5:1. D. Suction flow rate when using mucus with a water-to-mucin mixing ratio of 8:1.

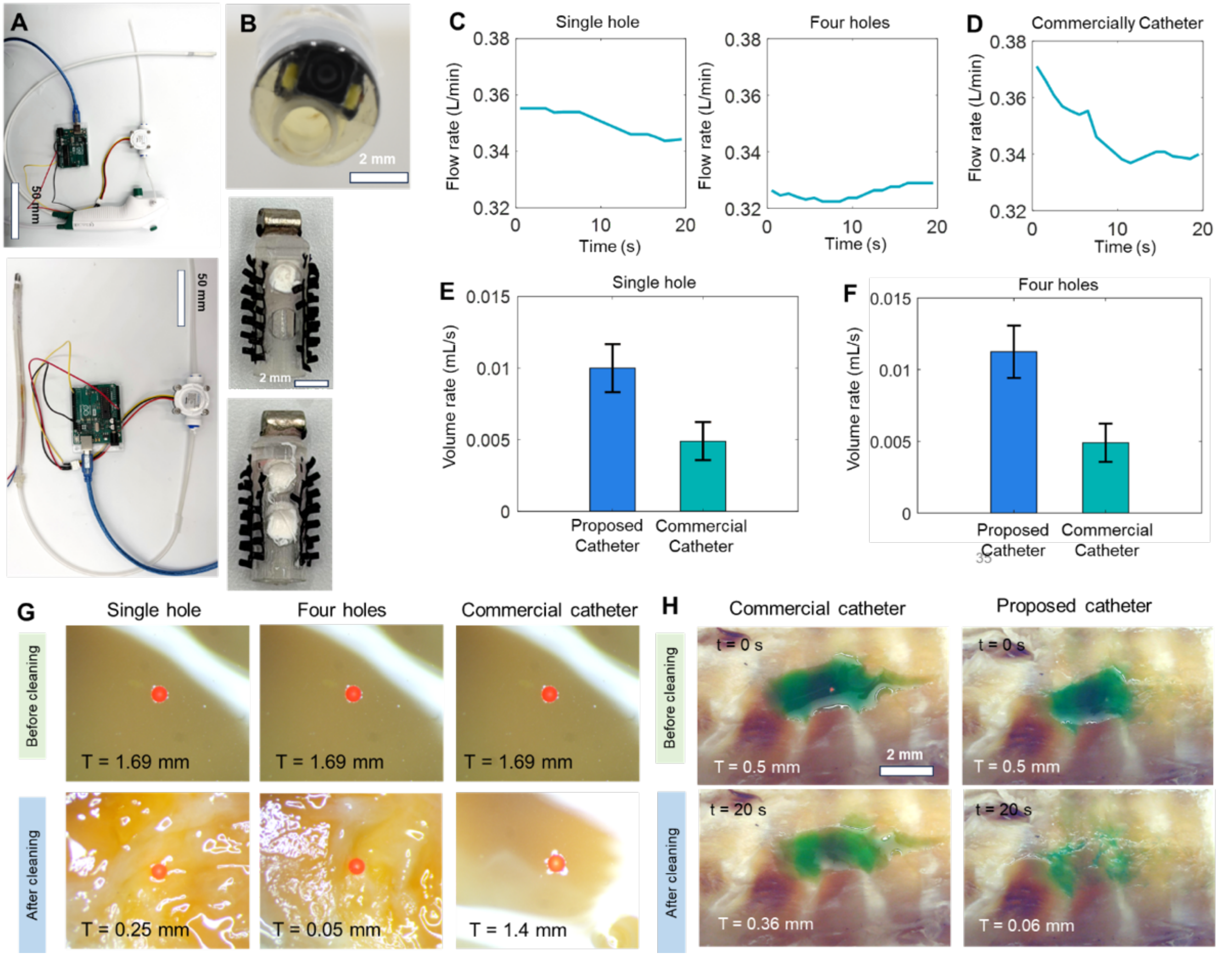

Fig. S6. Head-to-head comparison between the proposed soft ciliary catheter and a commercial suction catheter. A. Optical image of the experimental setup. B. Comparison of head tip areas between the two catheters. C. Flow rate comparison under identical suction conditions. D. Representative images showing mucus cleaning performance on tissue surfaces for both devices. E. Comparison of mucus removal volume rates for the two catheters with matched suction hole diameters. F. Comparison of mucus removal volume rates between the four-hole soft ciliary catheter and the single-hole commercial catheter. Error bars represent standard deviations (n = 5 trials). G. Measured mucus layer thickness after cleaning using the proposed catheter and the commercial catheter. The bead serves as a marker for determining mucus layer thickness. H. Quantitative comparison of overall mucus cleaning performance in terms of residual mucus thickness. Test conditions: mucin-to-water ratio = 1:5 (w/w); initial mucus layer thickness = 0.5 mm.

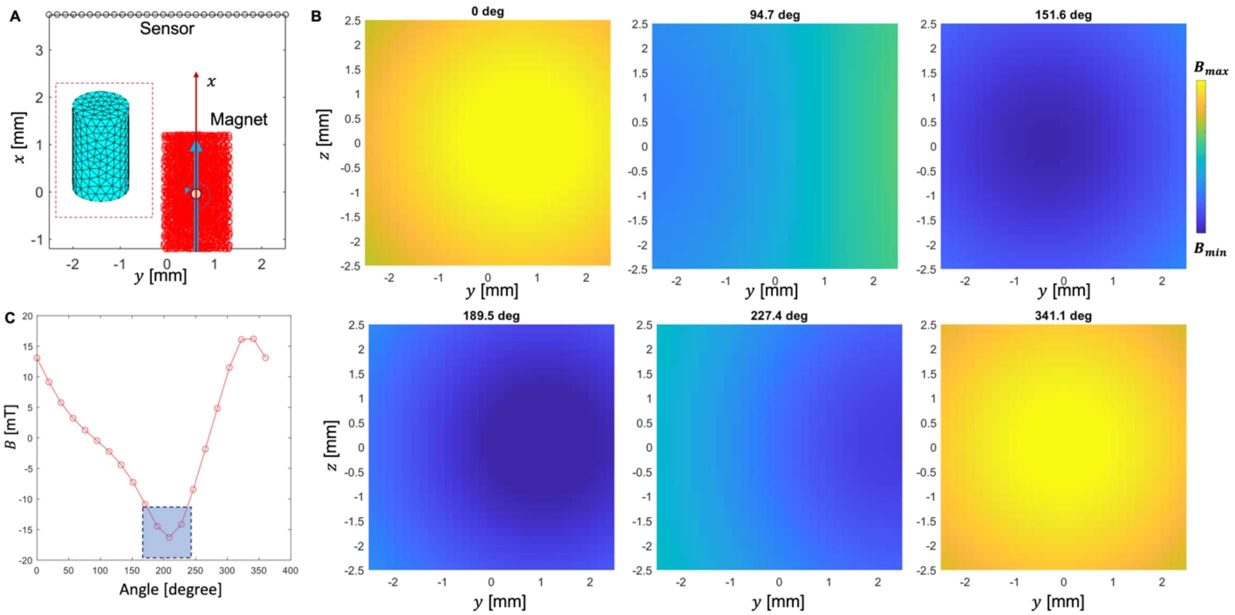

Fig. S7. Modeled magnetic field distribution in the sensor plane as a function of the cylinder magnet's rotational angle. (A) Illustration of the cylinder magnet with discretized elements, showing their relative positions and orientations with respect to the sensor. The magnet is offset along the y-axis due to the arrangement of the catheter cleaning head components. (B) Modeled B_x field distribution in the sensor plane (y–z plane). (C) Magnetic field at the sensor location (0, 0, 0) mm. The blue-shaded region indicates the range of magnet angles for which the x-direction field exceeds the sensor threshold, resulting in a low-value digital output.

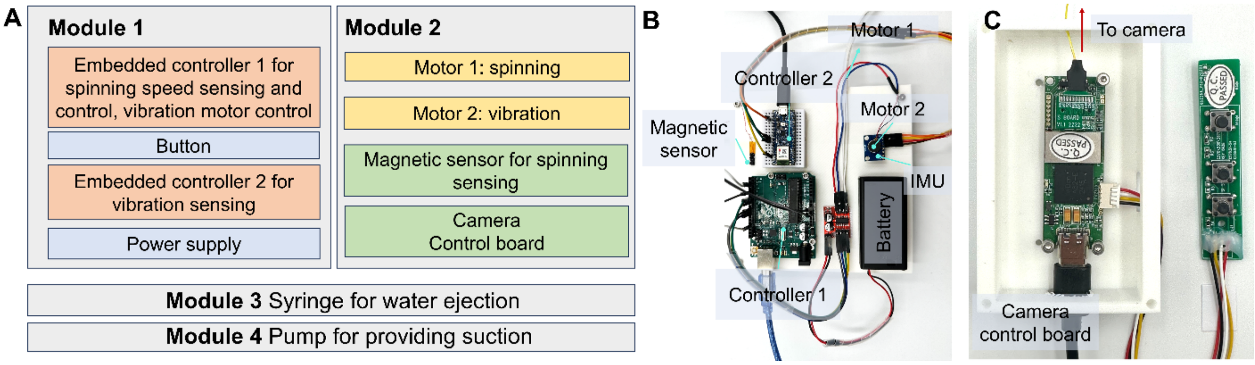

Fig. S8. System component diagram and optical images of the electronic boards. (A) System chart illustrating the modules of the catheter system: Module 1—embedded controllers and control interface; Module 2—motors, magnetic sensor, onboard camera, and its control board; Module 3—syringes for ejecting water to dilute viscous mucus and clean the camera lens; Module 4—pump for providing negative pressure for mucus suction. (B) Optical image of the motor control and haptic sensing board. Controller 1 connects to the magnetic sensor and regulates two motors: Motor 1 drives the spinning pump, and Motor 2 powers the vibration motor for user haptic feedback. Controller 2 connects the IMU for measuring the haptic feedback. (C) Optical image of the onboard camera control board, which connects to a personal computer for image processing.

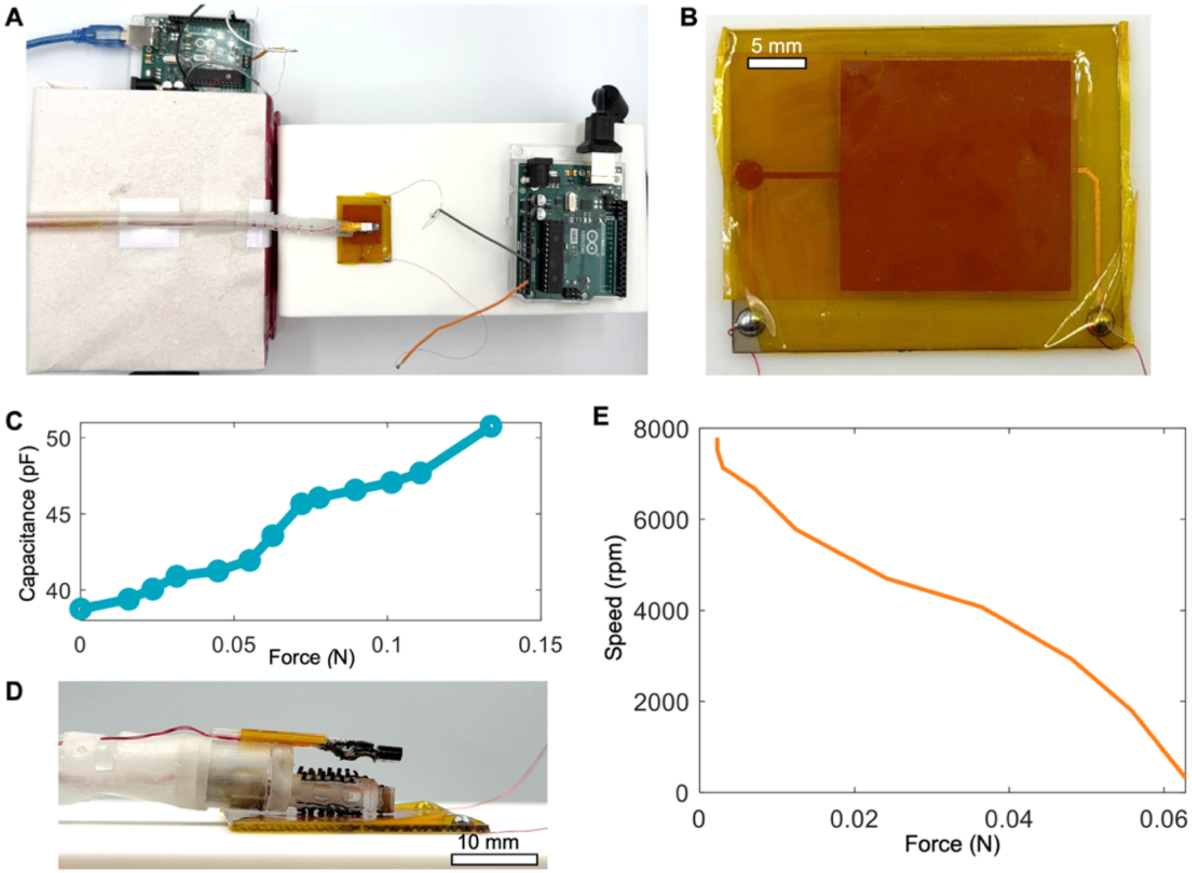

Fig. S9. Calibration of the contact force and rotational speed of the cleaning head during surface contact. A. Experimental setup for measuring contact force and cleaning head speed. B. Optical image of the soft dielectric force sensor composed of conductive plates and a dielectric polymer layer. C. Sensor capacitance as a function of applied force. D. Optical image showing the sensing process. E. Rotational speed of the cleaning head as a function of contact force.

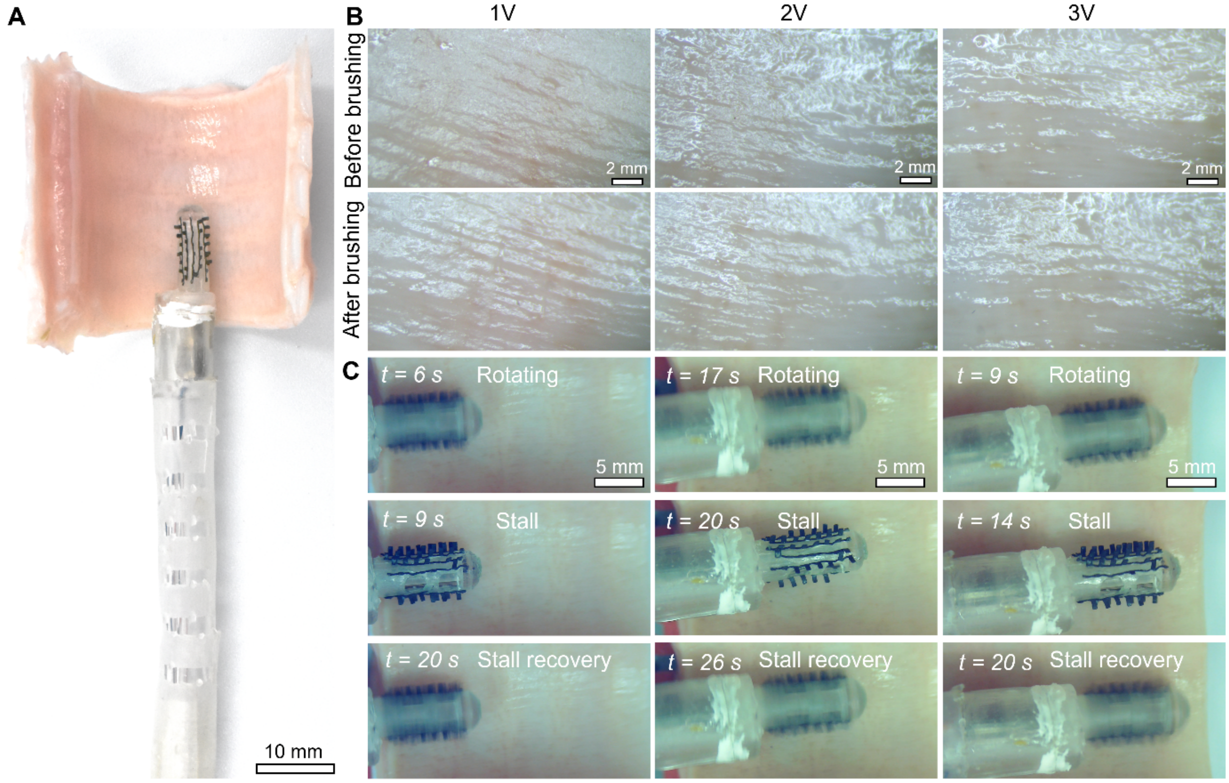

Fig. S10. Characterization of tissue response to the cleaning head at different driving voltages. A. Experimental setup for assessing potential tissue damage. B. Optical images of sheep trachea tissue before and after brushing at various driving voltages. C. Video frames illustrating the rotation, stall, and recovery phases of the spinning head during operation on sheep trachea tissue.

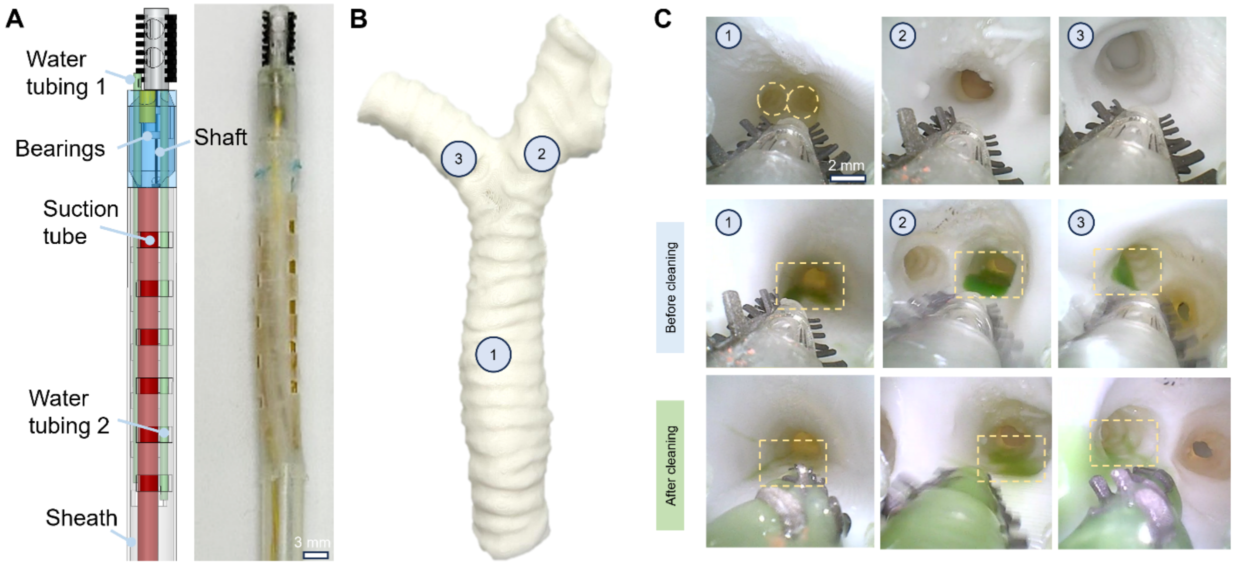

Fig. S11. Demonstration of a 6-mm-diameter catheter for mucus cleaning in the bronchi of a human airway phantom. (A) 3D rendering and optical image of the catheter with a 6-mm outer diameter. A 0.3-mm flexible nitinol shaft is used to drive the spinning pump. (B) Optical image of the human airway phantom of an adult male, showing three targeted locations. (C) Images from the onboard camera during catheter navigation and mucus cleaning.

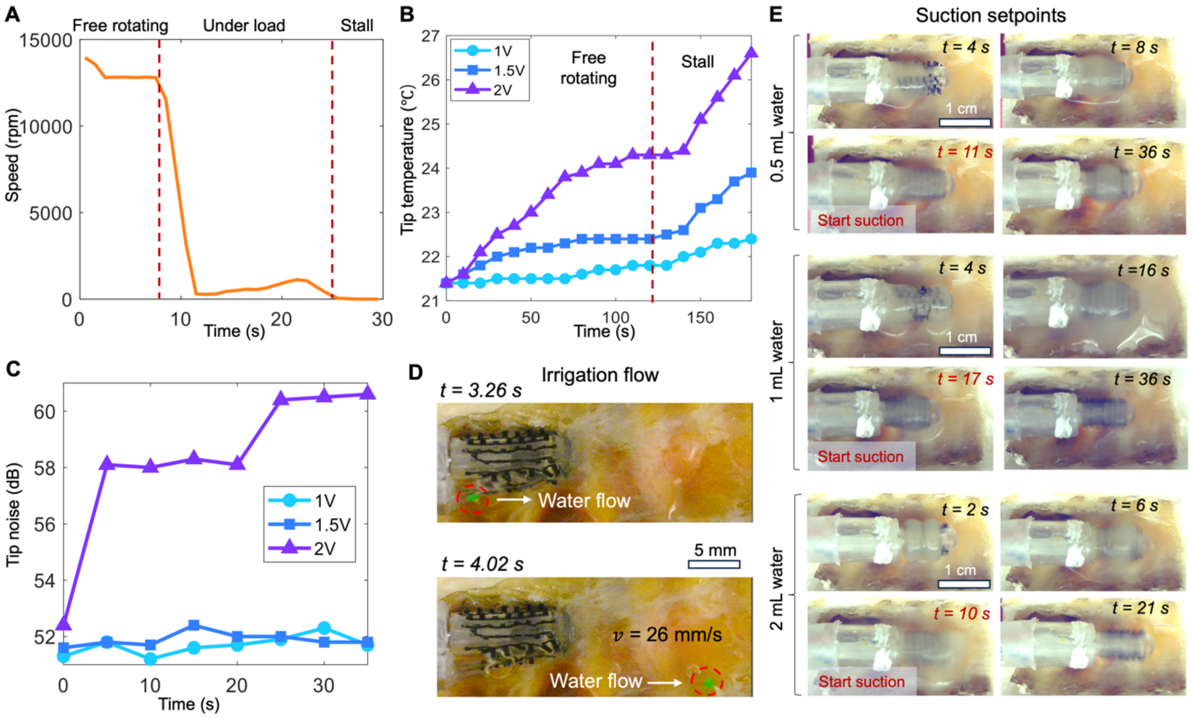

Fig. S12. Characterization of the catheter's spinning speed, noise, heat generation, and suction performance. A. Spinning head speed as a function of time. B. Tip temperature measured over time at different driving voltages. C. Acoustic noise levels of the tip under various driving voltages. D. Optical images showing irrigation flow speed measurements using plastic beads as tracers. E. Video frames illustrating suction setpoints in mucus samples after dilution with different volumes of water.

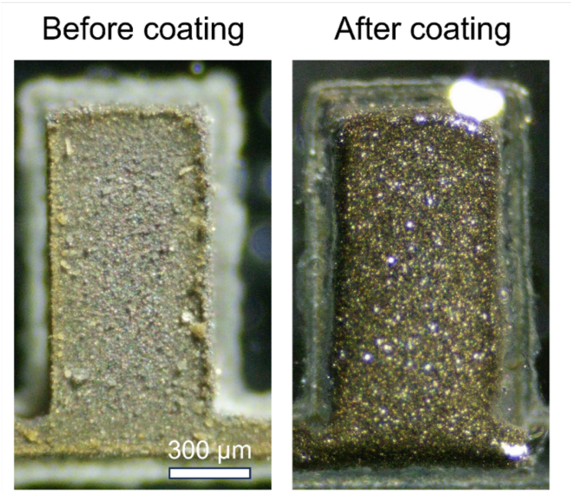

Fig. S13. Optical images of the magnetic artificial cilium before and after coating. The cilium is encapsulated with a thin layer of PDMS, and the embedded magnetic particles can be further coated with SiO_2 to enhance biocompatibility.

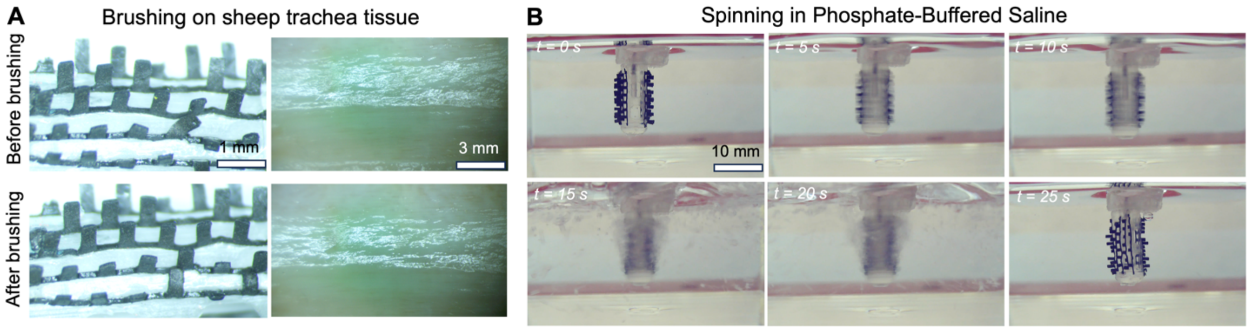

Fig. S14. Test of debris generation of the soft ciliary brush. A. Optical images of the soft ciliary brush and the biological tissue before and after the cleaning process. B. Video frames of the soft ciliary brush inside Phosphate-buffered saline after cleaning the tissue. No visible debris has been shown.

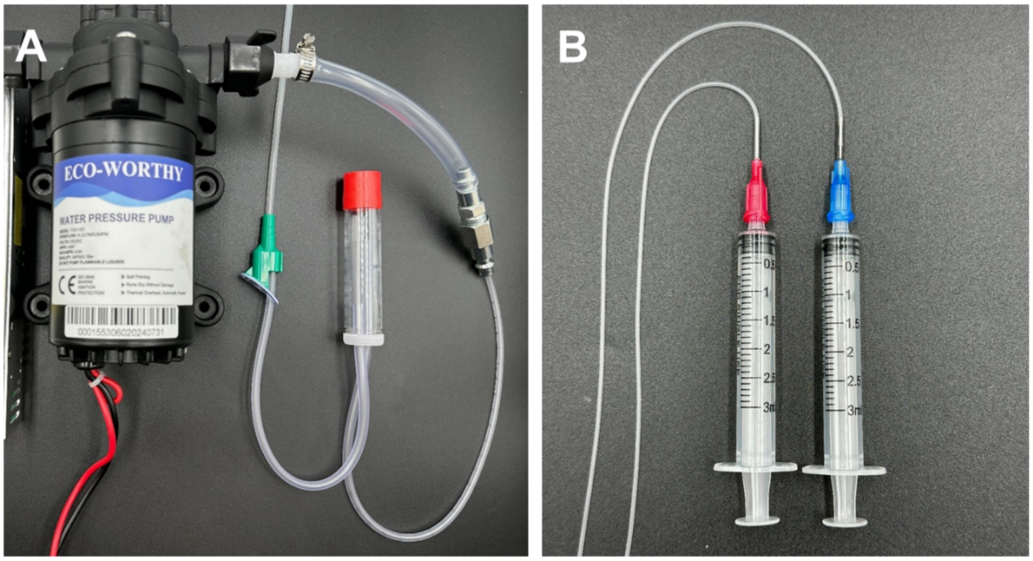

Fig. S15. Pumping units in the mucus cleaning system. (A) Optical image of the electrical pump used to generate negative pressure and the mucus collection container for storing removed mucus. (B) Optical images of two syringes for water ejection, used to dilute viscous mucus and clean the onboard camera lens.

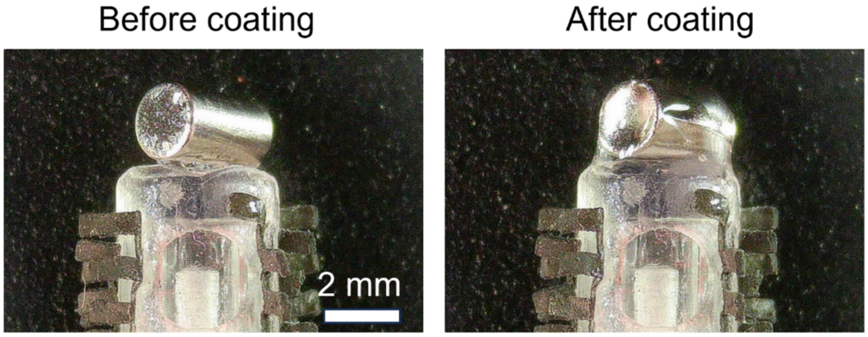

Fig. S16. Optical images of the magnetic marker before and after coating. The NdFeB magnet is encapsulated with a UV-curable resin layer to cover its surface and improve biocompatibility.

Table S1. Parameters of the motorized cleaning head

Name	Nominal value
**Overall dimension**	
Diameter of the catheter (motorized)	OD:8 mm, ID: 7mm
Length of the catheter	381 mm
Handle size	Length: 120 mm, OD: 30 mm
Steerable sheath	Length: 50 mm, Notch spacing: 2 mm
**Cleaning head**	
Spinner head diameter	OD: 4 mm, ID: 2.0-3.5 mm
Spinner head length	10 mm
Spinner hole size	Diameter: 2.5 mm
Artificial cilia array number	90
Artificial cilia size	Length: 1 mm, Width: 0.6 mm, Thickness: 0.25 mm
**Haptic unit dimension**	
Magnet size	OD: 1.5 mm, Length: 2.5 mm
Magnetic sensor size	Width: 4.1 mm, Thickness: 1.5 mm, Length: 3.2 mm
Vibration motor	OD: 8 mm, Thickness: 3 mm
**Other components**	
Water tubing diameters	OD: 1 mm ID: 0.5 mm
Camera diameter	OD: 2 mm

Table S2. Parameters of the flexible-shaft-based cleaning head

Name	Nominal value
**Overall dimension**	
Diameter of the catheter (motorized)	OD:6 mm, ID: 5.5 mm
Length of the catheter	381 mm
Handle size	Length: 120 mm, OD: 30 mm
Steerable sheath	Length: 50 mm, Notch spacing: 2 mm
**Cleaning head**	
Spinner head diameter	OD: 3 mm, ID: 1.5-2.5 mm
Spinner head length	10 mm
Bearing size	OD: 2 mm, ID: 1 mm.
Spinner hole size	Diameter: 2.5 mm
Flexible shaft	OD: 0.3 mm
Artificial cilia array number	46
Artificial cilia size	Length: 1 mm, Width: 0.6 mm, Thickness: 0.25 mm
**Haptic unit dimension**	
Magnet size	OD: 1.5 mm, Length: 2.5 mm
Magnetic sensor size	Width: 4.1 mm, Thickness: 1.5 mm, Length: 3.2 mm
Vibration motor	OD: 8 mm, Thickness: 3 mm
**Other components**	
Water tubing diameters	OD: 1 mm ID: 0.5 mm
Camera diameter	OD: 2 mm

Supplementary Note 1. Protocol for Head-to-Head Comparison Between Soft Ciliary Catheter and Therapeutic Bronchoscope

Objective:

To compare the mucus cleaning performance of the proposed soft ciliary catheter with a standard therapeutic bronchoscope under controlled and matched conditions.

Step 1: Device Preparation

1. Prepare the **soft ciliary catheter** prototype and a **therapeutic bronchoscope** for testing.
2. Integrate a **flow sensor** with each device to monitor suction flow rate in real time.
3. Configure both devices with a **single suction hole** of comparable diameter (~2.8 mm) to ensure equivalent inlet areas.

Step 2: Flow Rate Matching

1. Connect both devices to the **same suction pump setup** to standardize operating conditions.
2. Adjust the system to achieve **matched suction flow rates**, verified by flow sensor readings.

Step 3: Mucus Removal Tests

1. Apply mucus samples with controlled viscosity and layer thickness to the test surface.
2. Operate each device under identical suction conditions.
3. Measure the **mucus removal volume rate** for both devices.

Step 4: Performance with Multi-Hole Configuration

1. Test the **soft ciliary catheter** in its standard configuration featuring **four suction holes**, enabled by its larger cleaning head.
2. Observe how the **soft ciliary brush** directs mucus toward all inlets, maintaining stable suction pressure and preventing clogging.
3. Compare results with the **flat bronchoscope head**, which lacks lateral inlets and shows higher pressure drop.

Step 5: Quantitative Analysis

1. Quantify the **cleaning ratio** and **residual mucus layer thickness** after each cleaning trial.
2. Record and compare outcomes between the two devices.
    - The soft ciliary catheter shows a significantly **smaller residual layer** and higher cleaning efficiency.

Step 6: Failure Mode Assessment

1. Conduct additional trials using **thin mucus layers** to evaluate performance under challenging conditions.

2. Observe that the **commercial bronchoscope** struggles to remove thin mucus due to suction inefficiency and pressure drop.
3. Confirm that the **soft ciliary brush** maintains effective cleaning by wrapping and directing mucus toward the inlets, ensuring continuous removal.

SI Videos

Movie S1 Mechanism of mucus cleaning
This video shows mucus collection using only soft artificial cilia, only suction, and a combination of both.

Movie S2 Characterizing and optimizing the mucus cleaning performance
This video shows mucus collection by the cleaning head with varying hole sizes, cilia lengths, and cilia spacings.

Movie S3 Mechanism of sensing touch
This video demonstrates the mechanism for sensing spinner speed, captured through variations in the vibrating motor signal detected by an IMU.

Movie S4 Mucus cleaning performance with close-loop haptic feedback
This video demonstrates device steering in a phantom model for navigation, targeted mucus removal on ovine trachea tissue using both artificial cilia and suction, and real-time haptic feedback. It further showcases mucus cleaning in an *ex vivo* ovine lung, an airway stent, and an endotracheal tube.
